# Influence of Autochthonous Putative Probiotic Cultures on Microbiota, Lipid Components and Metabolome of Caciotta Cheese

**DOI:** 10.3389/fmicb.2020.583745

**Published:** 2020-10-21

**Authors:** Maria Barbara Pisano, Antonella Rosa, Danilo Putzu, Flaminia Cesare Marincola, Valentina Mossa, Silvia Viale, Maria Elisabetta Fadda, Sofia Cosentino

**Affiliations:** ^1^Department of Medical Sciences and Public Health, University of Cagliari, Cagliari, Italy; ^2^Department of Biomedical Sciences, University of Cagliari, Cagliari, Italy; ^3^Department of Chemical and Geological Sciences, University of Cagliari, Cagliari, Italy

**Keywords:** probiotic cheese, autochthonous cultures, lipids, metabolomics, NMR

## Abstract

The present study was undertaken to produce probiotic Caciotta cheeses from pasteurized ewes’ milk by using different combinations of autochthonous microbial cultures, containing putative probiotic strains, and evaluate their influence on gross composition, lipid components, sensory properties and microbiological and metabolite profiles of the cheeses throughout ripening process. A control cheese was produced using commercial starter cultures. The hydrophilic molecular pools (mainly composed by amino acids, organic acids, and carbohydrates) were characterized by means of ^1^H NMR spectroscopy, while the cholesterol, α-tocopherol and fatty acid composition by HPLC-DAD/ELSD techniques. Conventional culturing and a PCR-DGGE approach using total cheese DNA extracts were used to analyze cheese microbiota and monitor the presence and viability of starters and probiotic strains. Our findings showed no marked differences for gross composition, total lipids, total cholesterol, and fatty acid levels among all cheeses during ripening. Differently, the multivariate statistical analysis of NMR data highlighted significant variations in the cheese’ profiles both in terms of maturation time and strains combination. The use of autochthonous cultures and adjunct probiotic strains did not adversely affect acceptability of the cheeses. Higher levels of lactobacilli (viability of 10^8^–10^9^ cfu/g of cheese) were detected in cheeses made with the addition of probiotic autochthonous strains with respect to control cheese during the whole ripening period, suggesting the adequacy of Caciotta cheese as a carrier for probiotic bacteria delivery.

## Introduction

A “Functional Food” may be defined as any food with a positive impact on the consumer’s health, physical performance or state of mind in addition to its nutritious value (Karimi et al., 2012). The concept of biofunctional foods is generally used when the beneficial physiological effects are conferred by microorganisms (Gobbetti et al., 2010). These effects may derive from the interactions of ingested living microorganisms with the host (probiotic effect), and/or the ingestion of the microbial metabolites produced during fermentation (bioactive effect) (Linares et al., 2017). Lactic acid bacteria (LAB) play a multifunctional role in fermented foods that is not limited to the transformation of substrate and product preservation, but it is also related to the improvement of nutritional value and organoleptic characteristics as well as functional properties because of bioactive molecule production. LAB are generally considered beneficial microorganisms with some strains considered “probiotics.”

Probiotics, as part of functional foods, are a growing area of scientific interest for their role in maintaining a healthy intestinal ecosystem. They are bacteria, generally lactobacilli and bifidobacteria, but also enterococci and yeasts, associated with a plethora of health beneficial effects (Franz et al., 2011; Albenzio et al., 2013; Saad et al., 2013; Perotti et al., 2014; Merchàn et al., 2020). Probiotic food is a processed product which contains in a suitable matrix and in sufficient concentration viable probiotic microorganisms that must be able to survive in the gastrointestinal tract (Saxelin et al., 2003). Populations of at least 10^7^ CFU/g in the final product have been suggested as therapeutic quantities of probiotic cultures in different processed foods (Talwalkar and Kailasapathy, 2004; Karimi et al., 2012).

In the last decade, the interest in dairy products containing specific microbial strains with potential health-promoting properties has heightened. Cheese is one of the most common carriers used to efficiently deliver living probiotic microorganisms because it shows a good buffering capacity, generating a more favorable environment for probiotic survival throughout gastric transit (Castro et al., 2015; Homayouni et al., 2020). Several studies have addressed the development of probiotic cheeses including Cheddar (Zhang et al., 2013), Argentinean ovine cheese (Perotti et al., 2014) Scamorza (Albenzio et al., 2013) and Ricotta (Sameer et al., 2020).

Among the major challenges associated with the use of probiotic cultures for the production of functional cheeses are their ability to survive the technological hurdle during processing and ripening and their safety for human (Karimi et al., 2012; Castro et al., 2015). Furthermore, their introduction should not negatively affect the expected sensory characteristics (flavor, texture, and appearance) and biochemical composition of cheeses (Karimi et al., 2012; Sabikhi et al., 2014). In general, the incorporation of probiotic bacteria should not imply a loss of overall quality of the product.

*Lactobacillus* strains used as probiotics are generally of human or animal origin. However, several studies showed that strains with potential probiotic properties are also found in dairy products and other non-dairy fermented foods (Zielińska and Kolożyn-Krajewska, 2018). So far, many lactobacilli isolated from good quality artisanal raw milk cheeses have been selected on the basis of their functional properties and used along with standard or autochthonous starter cultures in the manufacturing process of different cheese types to improve and enhance their sensory quality (Randazzo et al., 2008; Guarrasia et al., 2017).

Presently, the use of probiotic yeasts is still scarce, despite their common presence in several fermented foods where they are actively involved in the production of aroma components and inhibition of spoilage bacteria (Fleet, 2007; Tofalo et al., 2019). On this basis, several yeast species have been extensively used as adjunct cultures to enhance flavor formation in certain cheese varieties (Centeno et al., 2017). The most important role of yeasts in cheese ripening is related to the increase of pH due to the metabolization of lactate and alkaline metabolite formation.

The technological, probiotic and bioactive potential of these putative probiotic strains should be evaluated *in situ* by analyzing their interactions in terms of cheese composition, physical chemical characteristics, nutrient availability, viability and biomolecules production.

Caciotta is as a semi-soft cheese with a short-medium ripening time produced from pasteurized whole cow’s, ewe’s or goat’s milk, and represent one of the most common traditional Italian cheese variety. “Caciotta sarda” is produced from December to July throughout the Sardinian territory using Sardinian sheep’s milk. The shape is cylindrical and the weight varies from 1 to 2.2 Kg. The milk is pasteurized, cooled to 36°C and coagulated with the addition of calf rennet and commercial microbial starter.

The organoleptic characteristics of this cheese type can vary as a function of the production area, the ripening time and the milk used (Gobbetti et al., 2018). Since the ripening time generally varies from 15–20 days to 2–6 months, milk pasteurization is needed to eliminate any pathogenic bacteria possibly present in raw milk (Aquilanti et al., 2011). On the other hand, pasteurization causes the loss of indigenous LAB species relevant for fermentation and ripening process. Therefore, in order to standardize the production while preserving at the same time the unique characteristics of this typical cheese variety, the addition of selected autochthonous adjunct cultures appears as a most efficient tool, as reported in other studies (Fusco et al., 2019; Bancalari et al., 2020; Calasso et al., 2020). The use of autochthonous starter cultures has already been proved to be an effective mean for standardizing the production of raw milk cheeses at the same time positively affecting their quality (Silvetti et al., 2017; De Pasquale et al., 2019; Dolci et al., 2020). When applying pasteurization to reduce cheese defects, the addition of selected LAB strains is of particular relevance for reconstituting the original microflora responsible for the typicality of traditional products, as shown by other authors for Caciotta cheese (Turchi et al., 2011; Bancalari et al., 2020).

In this work, we used a multidisciplinary approach to investigate the influence of adjunct cultures, containing autochthonous putative probiotic *Lactobacillus* and yeast strains, on the chemical and microbiological composition and sensory properties of industrially produced ewe’s milk Caciotta cheese throughout a ripening period of 60 days. For the sake of comparison all analyses were performed also on Caciotta made with commercial starter cultures used as control.

## Materials and Methods

### Microbial Strains and Cheese Manufacture

As reported in Table 1, three types of experimental Caciotta cheeses (C1, C2, C3) were made using three different combinations of autochthonous adjunct cultures, containing putative probiotic *Lactobacillus* and yeast strains coupled with starter cultures represented by autochthonous *L. lactis* subsp. *lactis* or *Enterococcus faecalis* strains, selected on the basis of their technological characteristics and safety properties needed for the application as functional starter cultures (Cosentino et al., 2002, 2004; Pisano et al., 2015). The *Lactobacillus* and yeasts strains used in this study were previously tested for some *in vitro* functional properties associated with probiotic features in view of their use in cheese production as adjunct cultures (Pisano et al., 2008, 2014; Fadda et al., 2004, 2017). The main features of the autochthonous strains used in this study have been reported in Supplementary Table 1 in Supplementary Material. A control cheese (CC) was produced using a commercial starter culture (*L. lactis* Lyoto MO540 and MO536, Sacco, Italy). All cheeses were manufactured by a dairy plant (Argiolas Formaggi, Dolianova, Cagliari, Italy) according to their Caciotta production protocol.

**TABLE 1:** Starter and putative probiotic cultures used in the manufacturing of ovine Caciotta cheeses.

**Caciotta types**	**Cultures**	**Origin**
CC	*Lactococcus lactis subsp. lactis* Lyoto MO540	Commercial starter cultures
	*Lactococcus lactis subsp. lactis* Lyoto MO536	Commercial starter cultures
C1	*Lactococcus lactis subsp. lactis* 6MRSLS5	Raw sheep milk
	*Lactobacillus plantarum* 19/20711	Raw sheep milk
	*Debaryomyces hansenii* (FS6 DH1)	Fiore Sardo cheese
C2	*Lactococcus lactis subsp. lactis* 1FS171M	Fiore Sardo cheese
	*Lactobacillus plantarum* 62LP39b	Raw sheep milk
C3	*Enterococcus faecalis* 3M17LS5	Raw sheep milk
	*Lactobacillus plantarum* 11/20966	Raw sheep milk
	*Kluyveromyces lactis* 17bKL2	Fiore Sardo cheese

The autochthonous LAB strains were maintained at −20°C in MRS or M17 broth (Microbiol, Cagliari, Italy) with sterile 15% (v/v) glycerol (Microbiol) as cryoprotector in the microbial collection of the Department of Medical Sciences and Public Health. Fresh cultures were prepared in autoclaved sterile skimmed milk after two consecutive transfers in MRS or M17 broth (1% inoculum) incubated at 30°C in aerobic conditions for 18 h. Yeasts strains were maintained in YEPD (Yeast Extract Peptone Dextrose, Microbiol) with 20% (w/v) glycerol. The strains used in combination did not produce antibacterial substance against each other (data not shown).

Overall, four Caciotta cheese-making trials were carried out in four different days. In each trial two cheese batches were simultaneously produced from two vats of the same pasteurized ewes’ milk using two different starter combinations (commercial starter or autochthonous starter containing putative probiotic strains as adjunct) for a total of eight cheeses batches, so two independent experiments were made in different days for each strains combination (Supplementary Figure 1 in Supplementary Material). To prepare the inoculum for cheese making, the strains were inoculated in pasteurized ovine milk incubated at 30°C for 18–24 h and added to vat milk at a level of 10^7^, 10^5^ and 10^4^ cfu/mL for LAB, *Kluyveromyces* and *Debaryomyces* species, respectively.

The chemical composition of raw ewe’s milk used for cheese-making was 6.43% fat, 5.58% protein, 4% lactose, and the pH measured at 6.7. After pasteurization (72°C for 20 s), milk in each vat (150 L) was cooled at 37°C and inoculated with starter and adjunct cultures. After 30 min of resting time, rennet was added to the milk (1:50000) and coagulation took place at 37°C within 20 min. The curds were cut and left to rest for 10 min. Then, the curd pieces were hand pressed into molds for whey drainage (30°C, 85–90% of humidity). After brine salting for 12–16 h (23% °Be), the cheeses were transferred to a ripening chamber and stored 2 months at 12–15°C and 85% relative humidity. The weight of the cheeses was about 1.5 kg, diameter was 20 cm, and height was 15 cm. Samples were taken for all analysis at 1, 15, 30, and 60 days after production. The cheeses were sent to the laboratory under refrigeration (ca. 4°C) and were either analyzed immediately or frozen, depending on the assays.

### Compositional Analysis

Chemical composition (fat (wet weight), fat (dry weight), protein, salt and moisture) was determined by a FoodScan^TM^ Lab Dairy Analyser type 78810 (Foss, Hillerød, Denmark) by the dairy plant laboratory. The pH of the milk and the homogenized cheese samples (10 g in 10 ml of distilled water) was measured with a HI8520 pH meter (Pool Bioanalysis Italiana, Milan, Italy). Water activity (a_w_) was determined using a PAWKIT water activity meter (Decagon Devices, Pullman, WA, United States) in accordance with the manufacturer’s instructions. All determinations were made in duplicate.

### Microbiological Analyses

At each sampling point a slice from a full piece of cheese (ca. 200 g) was sampled, then triplicate aliquots of 10 g were added to 90 mL of 2% (w/v) sodium-citrate sterile solution and homogenized in a Stomacher Laboratory Blender (Pool Bioanalysis Italiana, Milan, Italy) for 2 min at normal speed. Decimal dilutions were prepared in sterile 0.1% (w/v) peptone solution and spread onto the surface of the different agar media. Total mesophilic aerobic microbiota was enumerated using the pour plate method on Plate Count agar (PCA, Microbiol, Cagliari, Italy) incubated at 30°C for 48–72 h. *Enterobacteriaceae* and *E. coli* were determined on Violet Red Bile Glucose agar (VRBGA, Microbiol) and Triptone Bile X-gluc agar (TBX, Microbiol), respectively, using the pour plate method. VRBG and TBX plates were covered with a layer of the same culture medium before incubation at 37°C and 44°C for 24 h, respectively. Lactococci and enterococci were counted in M17 and KF agar (Microbiol) supplemented with 1% TTC, incubated at 30 and 37°C for 48 h, respectively. For the enumeration of lactobacilli, MRS agar acidified at pH 5.4 with glacial acetic acid incubated at 30°C in microaerophilic conditions for 48 h was used. Coagulase positive staphylococci were counted on Baird-Parker egg-yolk-tellurite agar (Microbiol) incubated at 37°C for 48 h. Black colonies with or without the typical clearing of the egg yolk were tested for catalase-positivity, mannitol fermentation and coagulase production by the tube coagulase test. Yeasts and molds were enumerated in Potato Dextrose agar (PDA, Microbiol) plates containing 0.1 g/L cloramphenicol incubated at 25°C for 5 to 20 days.

### DNA Extraction From Cheese Samples

Total DNA was extracted directly from samples collected during cheese making and ripening. At each sampling point a slice from a full piece of cheese (ca. 200 g) was sampled and a 25 g aliquot was homogenized in 225 mL of sodium citrate (pH 7.5), using a Stomacher for 3 min, then 1 mL of the homogenate was incubated for 30 min at 45°C in a dry Thermo-block heater (Asal, Milan, Italy). After the addition of equal volume of absolute ethanol, the samples were centrifuged at 10000 × *g* for ten min. The pellets were resuspended in 200 μL lysis buffer (200 mM Tris–HCl, pH 8.0, 250 mM NaCl, 25 mM EDTA, SDS 0.5% w/v) and 18 μL of proteinase K solution (2 mg/mL, Sigma) were added. The sample was vigorously vortexed and incubated for 60 min at 65°C before addition of 20 μL 2.5 M potassium acetate (Sigma). The resulting mixture was placed on ice for 2 h before centrifugation at 12.000*g* for 30 min. Two hundred microliters of the supernatant were transferred to a new tube and an equal volume of 2-propanol (Sigma) was added to precipitate nucleic acids. Nucleic acids were extracted in the aqueous phase by centrifugation at 12000 × *g* (5′, 4 °C) by adding 750 μL of SEVAG (Chloroform: isoamilic alcohol 24:1). The resulting DNA solution was precipitated with 70% ethanol by centrifugation at 12000 (4°C, 30 min), air-dried and resuspended in 100 μL of TE (10 mMTris–Cl, pH 8.0, 1 mM EDTA). DNA concentration and purity were checked by optical density at 260 nm and ratio O.D. 260 nm/280 nm determinations, respectively.

### PCR Amplification of 16S and 26S rDNA Sequences and DGGE Analyses

For DGGE analysis, the universal primers F357 (5′-TACGGGAGGCAGCAG -3′) with the GC clump and R 518 (5′-ATTACCGCGGCTGCTGG-3′) (Muyzer et al., 1993) were used to amplify the V3 region of 16S rRNA. Yeast DNA amplifications were carried out using primers NL1 (5′-GCA TAT CAA TAA GCG GAG GAA AAG-3′), with the GC clamp and LS2 (5′-ATT CCC AAA CAA CTC GAC TC-3′) (Cocolin et al., 2000). Amplification reactions were performed in a Mastercycler gradient 5331 (Eppendorf) in a final 50 μL reaction mixture volume each containing 1 × PCR buffer, 2.5 mM MgCl_2,_ 200 μM dNTPs, 0.2 mM primer, 2.5 U of *Taq* DNA polymerase (Sigma), and 10 μL of template DNA. PCR products were routinely checked for positive amplification on 1.8% w/v agarose gels prior to further DGGE analysis.

PCR products were analyzed by DGGE using a Bio-Rad D-code^TM^ apparatus (Bio-Rad Laboratories) on 20 cm × 20 cm × 1 mm gel. Parallel electrophoresis was performed in 1 × TAE at 60 °C, employing 8% polyacrylamide gels with a denaturing range of 40–60%. The gels were run for 16 h at 75 V. Bands were visualized under UV light after staining with ethidium bromide (0.5 mg mL^–1^), and photographed under UV trans-illumination. A ladder consisting of an amplicon mix of the different species used as starter and adjunct probiotic cultures in the manufacturing of caciotta cheeses was created in order to monitor their presence throughout ripening.

### Sensory Analysis

Descriptive sensory profile of 60 days-ripened cheeses was carried out by a panel of six assessors trained in the sensory analysis of Caciotta cheeses produced in Sardinia. The experimental and control cheeses were evaluated for their general appearance, odor intensity, taste (acid, sweet, bitter, salty) aroma intensity, texture (intensity of elasticity, firmness, solubility, granularity) and overall acceptability, using 7-point category scale, ranging from 1 (low or poor) to 7 (high or excellent). The panelist had no information about the experimental design. All cheeses were kept at 20°C for 1 h before starting the test, coded with a three-digit code number and presented in random order.

### Extraction and Saponification of Caciotta Cheeses Lipids

Total lipids (TL) were extracted from portions of grated cheese samples (100 mg, in triplicate) by the Folch procedure (Folch et al., 1957), using the mixture chloroform:methanol:water 2:1:1 (v/v/v) as previously reported (Rosa et al., 2015). The chloroform fraction (lipid extract) from each sample was separated from the methanol/water mixture and dried down under vacuum. The dried lipid fractions, dissolved in ethanol, were subjected to mild saponification as previously reported (Rosa et al., 2015, 2017) for the separation of lipid components. The unsaponifiable (total cholesterol and α-tocopherol) and saponifiable (FA) fractions were collected, the solvent evaporated, and a portion of the dried residues was injected into the high-performance liquid chromatograph (HPLC) (Rosa et al., 2015, 2017). An external standard mixture (containing 1 mg of triolein, trilinolein, and cholesterol) was processed as samples to calculate the fatty acid and cholesterol recovery during saponification.

### Analyses of Fatty Acids, Cholesterol, and α-Tocopherol in Caciotta Cheeses

Analytical standards of FA, cholesterol, trilinolein, triolein, α-tocopherol, conjugated (9Z,11E)-linoleic acid (CLA), and all solvents used, of high purity, were purchased from Sigma–Aldrich (Milan, Italy). All the chemicals used in this study were of analytical grade.

Analyses of lipid components were carried out with a 1100 liquid chromatograph (Agilent Technologies, Palo Alto, CA, United States) equipped with a diode array detector (DAD) and an Infinity 1260 evaporative light scattering detector (ELSD). Total cholesterol (detected at wavelength of 203 nm) and α-tocopherol (at 292 nm) were measured with an Inertsil ODS-2 column and methanol as the mobile phase (at flow rate of 0.7 mL/min) as previously reported (Rosa et al., 2015, 2017). Analyses of FA (unsaturated were detected at 200 nm and with ELSD, saturated with ELSD) were carried out with a XDB-C_18_ Eclipse, with a mobile phase of acetonitrile/water/acetic acid (75/25/0.12, v/v/v), at a flow rate of 2.3 mL/min as previously reported (Rosa et al., 2015, 2017). The temperature of the column was maintained at 37°C. ELSD detector settings were: evaporator temperature 40°C, nebulization temperature 40°C, with nitrogen as the nebulizing gas at a flow of 1 L/min. The FA identification and quantification were performed using standard compounds and the Agilent OpenLAB Chromatography data system as previously described (Rosa et al., 2015, 2017). Calibration curves of the lipid compounds were found to be linear (DAD) and quadratic (ELSD), with correlation coefficients > 0.995.

### NMR Analysis

A total of 96 samples were prepared for metabolomics analysis (2 trials × 4 added culture mixtures × 4 time points × 3 replicates). At each ripening time, a slice of cheese (ca. 300 mg) was sampled. Cheese was removed from 1 cm of the crust, freeze dried and then powdered in a ceramic mortar with a pestle. A methanol-chloroform-water extraction was performed for each cheese sample in triplicate as previously reported (Piras et al., 2013). The methanol/water fraction was separately collected, dried in vacuum at room temperature by using an Eppendorf concentrator 5301 (Eppendorf AG, Hamburg, Germany), and then stored at −80 °C. Prior to ^1^H NMR analysis, the hydrophilic metabolites were dissolved in 700 μL of a D_2_O solution containing 0.80 mM sodium 3-trimethylsilyl-(2,2,3,3-d4)-1-propionate (TSP) used as internal standard. The pH of the final sample was adjusted to 4.00 ± 0.05 by adding small amounts of NaOD or DCl. Then, an aliquot of 650 μL was transferred into a 5 mm NMR tube.

^1^H NMR spectra were recorded on a Varian UNITY INOVA 500 spectrometer operating at 499.839 MHz. Experiments were carried out using the standard Varian PRESAT pulse sequence. For each experiment, 256 scans were collected into 32 k data points at 300 K over a spectral width of 6000.6 Hz with a 90° pulse, an acquisition time of 2.5 s, and a relaxation delay of 4 s. A presaturation technique with low power radiofrequency irradiation for 2 s was applied to suppress the residual water signal. The FIDs were multiplied by an exponential weighting function equivalent to line broadening of 0.3 Hz prior to Fourier transformation. ^1^H NMR chemical shifts were referenced to TSP (δ 0.0 ppm). The assignment of NMR peaks was performed according to literature data (Piras et al., 2013), the Food Database,^1^ and Chenomx NMR suite 8.1 software (evaluation version, Chenomx, Edmonton, AB, Canada).

All spectra were phased and baseline corrected using MestReNova (Version 14.0, Mestrelab Research SL, Santiago de Compostela, Spain). Correction for minimal misalignments in chemical shift, mainly due to pH-dependent signals, was done before deleting the regions containing the residual water and internal standard TSP signals, and segmenting the NMR spectra into intervals (bins) with equal width of 0.0025 ppm over a chemical shift range of 0.5–9.5 ppm. Bins were then normalized to the sum of total spectral area to compensate for the overall concentration differences.

### Statistical Analyses

Data on compositional, microbiological and sensory analyses were analyzed using the software GraphPad Prism Statistics software package version 5.00 (GraphPad Prism Software Inc., San Diego, CA, United States). Means were compared by one-way analysis of variance (One-way ANOVA) using Bonferroni Multiple Comparisons Test. The data on sensory analyses were analyzed by Kruskal-Wallis test in order to detect any significant differences between the organoleptic profiles of the cheeses. A difference of *P* < 0.05 was considered significant.

Multivariate statistical analysis (MVA) of the NMR-data set was conducted by using SIMCA version 16.0 (Umetrics, Umea, Sweden). Prior to MVA, data were Pareto scaled. An explorative principal component analysis (PCA) was first performed to overview the variability of data and possible trends of groups (Bro and Smilde, 2014). The performance of the PCA model was evaluated using the coefficients *R*^2^ and *Q*^2^, defined as the proportion of variance in the data explained and predictable by the model, respectively. To investigate the relationship between the Nuclear magnetic resonance (NMR) spectral profile of cheeses and the ripening time, orthogonal partial least squares (OPLS) was applied (Trygg and Wold, 2002). OPLS is an extension of PLS that splits the systematic variation in the X block into two parts, one that models the correlation between X and Y (predictive) and another that shows the systematic X variation not related (orthogonal) to Y. The complexity of the model is reduced by orthogonalization since the systematic variation in the X matrix not correlated with Y is removed. Orthogonal projections to latent structure-discriminant analysis (OPLS-DA) was employed in a pair-wise comparison to discriminate groups (classes) of cheeses at 60 days of ripening (Bylesjo et al., 2006). The quality of OPLS and OPLS-DA models was evaluated by *R*^2^Y and *Q*^2^Y, i.e. the fraction of the variation of Y-variable and the predicted fraction of the variation of Y-variable, respectively. Permutation test was used to assess the reliability of models and CV-ANOVA (Cross-Validation Analysis Of Variance) test to evaluate their significance. Potential variables that are statistically significant were selected by analyzing the S-line correlation coefficient plot that is tailor-made for NMR spectroscopy data: peaks with positive (negative) intensity belong to the most abundant metabolites in the group on the right (left) side of the scores plot; peak in warm colors belong to metabolites that contribute more significantly to the class separation than do the metabolites associated with signals in cool colors. Variables were selected according to a *p*(corr) ≥|0.5| and *p*(cov) ≥|0.05| The correlation coefficient *p*(corr) value refers to the credibility of the contribution of the variable in the mathematical model, while *p*(cov) represent the modeled covariation.

## Results and Discussion

### Physico-Chemical Composition of Caciotta Cheese During Ripening

The physico-chemical compositions (pH, a_w_, moisture, fat, fat DM, salt, protein) of experimental and control Caciotta cheeses at 1, 15, 30, and 60 days of ripening are reported in Table 2. The pH evolution over the 60 day of ripening varied among cheese types: the mean values initially decreased up to 15 (in C1 and C3) or 30 (in CC and C2) days of ripening, then increased at 60 days in all cheeses. Although no significant differences were observed among cheese types, a faster increase in pH (from 30 to 60 days of ripening) was recorded in cheeses produced with adjunct probiotic strains compared to the control (ΔpH CC: 0.1; ΔpH C1, C2, C3: 0.41, 0.69, 0.91, respectively). The highest pH value observed in C3 at the end of maturation could be related to both the proteolysis during ripening and the lactic acid utilization by the yeasts strain *K. lactis* used as adjunct in the manufacturing of the cheese (Fadda et al., 2004). The mean water activity (a_w_) of all cheeses decreased during ripening, achieving values around 0.94–0.96 at 60 days, with no significant differences (*P* > 0.05) among the batches. A_w_ is a key factor in maintaining probiotic viability in the final products and the values observed in our study are unlikely to affect the viability and growth of the potential probiotic strains, since *Lactobacillus*, *Debaryomyces hansenii* and *Kluyveromyces lactis* species have been shown to possess stability in low water activity and maintain their viability in different low moisture foods (Tapia et al., 2007; Marcial-Coba et al., 2019).

**TABLE 2:** Physico-chemical characteristics of ovine Caciotta cheeses during ripening.

**Parameter**	**Caciotta type**	**Ripening time (days)**			
		**1**	**15**	**30**	**60**
pH	CC	5.28 ± 0.41	4.92 ± 0.42	4.90 ± 0.31	5.07 ± 0.15
	C1	6.06 ± 0.39	5.10 ± 0.40	5.12 ± 0.39	5.59 ± 0.80
	C2	5.30 ± 0.37	5.02 ± 0.31	4.97 ± 0.25	5.66 ± 0.99
	C3	5.82 ± 0.57	4.81 ± 0.08	4.88 ± 0.09	5.80 ± 0.57
a_w_	CC	0.99 ± 0.01	0.98 ± 0.00	0.98 ± 0.01	0.96 ± 0.00
	C1	0.990,01	0.97 ± 0.01	0.97 ± 0.00	0.96 ± 0.01
	C2	0.990,02	0.97 ± 0.00	0.97 ± 0.00	0.94 ± 0.02
	C3	1.000,02	0.98 ± 0.01	0.97 ± 0.02	0.95 ± 0.01
Moisture (%)	CC	49.68 ± 2.26	47.28 ± 2.24	46.041.03	40.38 ± 2.45
	C1	50.84 ± 0.11	47.34 ± 2.90	46.96± 4.17	43.64 ± 2.99
	C2	47.71 ± 1.24	44.58 ± 2.45	44.26 ± 2.46	37.94 ± 4.03
	C3	49.74 ± 0.92	48.87 ± 0.06	46.37 ± 2.76	43.67 ± 2.37
Fat (%)	CC	25.16 ± 0.25	26.50 ± 0.17	27.14 ± 0.90	31.18 ± 2.33
	C1	24.12 ± 0.79	26.03 ± 2.01	26.69 ± 2.23	28.59 ± 2.11
	C2	26.58 ± 1.90	28.63 ± 2.67	30.555 ± 3.86	32.66 ± 6.15
	C3	25.06 ± 1.48	24.65 ± 0.07	26.41 ± 2.67	27.85 ± 2.26
Fat (%DM)	CC	50.06 ± 2.75	50.32 ± 2.47	50.31 ± 2.64	51.20 ± 3.25
	C1	49.07 ± 1.70	49.40 ± 1.10	49.81 ± 0.94	50.23 ± 0.38
	C2	50.82 ± 2.43	51.62 ± 2.52	52.82 ± 4.43	53.91 ± 4.40
	C3	49.85 ± 2.05	48.20 ± 0.08	49.18 ± 2.45	49.39 ± 1.93
Salt (%)	CC	1.02 ± 0.71	1.27 ± 0.52	1.65 ± 0.42	2.06 ± 0.03
	C1	1.32 ± 0.29	1.54 ± 0.10	1.76 ± 0.08	2.24 ± 0.16
	C2	1.32 ± 0.29	1.54 ± 0.10	1.76 ± 0.08	2.24 ± 0.16
	C3	0.95 ± 0.17	1.55 ± 0.09	1.80 ± 0.02	2.19 ± 0.25
Protein (%)	CC	20.93 ± 0.42	21.77 ± 0.09	22.15 ± 0.37	24.851.56
	C1	19.86 ± 0.52	21.60 ± 1.32	21.71 ± 1.48	22.96 ± 1.01
	C2	21.02 ± 0.03	21.87 ± 0.08	22.54 ± 0.71	24.14 ± 0.56
	C3	20.99 ± 0.80	21.20 ± 0.05	21,95 ± 1.27	23.62 ± 1.31

*Data are the mean ± standard deviation (SD) of two cheese-batches. No significant differences (*P* > 0.05) were detected among cheese types for all parameters analyzed.*

With regard to the compositional parameters, no significant differences (*P* > 0.05) were detected between the control and the experimental cheeses. The mean values of fat, protein and salt increased during ripening, coupled with a decrease in the moisture mean content in all cheese types, in agreement with previous results (Pisano et al., 2007; Buccioni et al., 2012). At day 60, the composition of cheeses was found within the range of other Italian ewes’ milk cheeses (Coda et al., 2006). It has been previously observed that the addition of probiotic microorganisms did not affect the main compositional parameters (moisture, salt, fat and protein contents) of different cheese types (Zhang et al., 2013; Perotti et al., 2014; Terpou et al., 2018), and did not negatively influence the ripening process of Edam cheese (Sabikhi et al., 2014).

### Microbiota Analysis

In all the analyzed cheese samples, the overall hygienic quality was evaluated by the determination of microbial hygiene indicators *Enterobacteriaceae* and coagulase positive staphylococci, which were always under the detection limit (< 10 cfu/g and < 100 cfu/g, respectively) (data not shown). It has been demonstrated that efficient starter culture and/or an enforcement by an adjunct is essential to achieve a fast pH decrease, thus contributing to control these undesired microorganisms (Terpou et al., 2018).

The viable counts of LAB and yeasts are shown in Table 3. The numbers of presumptive lactococci were high throughout the ripening and did not significantly differ (*P* > 0.05) among all samples, presumably due to the use of the same type and amount of starter cultures. The presence of lactococci in C3 cheeses (where no lactococcal strain was included in the starter) may be due to secondary contamination considering that they are widely used as starter in cheese productions and taking into account their ability to colonize and adapt to different substrates in the dairy plant (Calasso et al., 2016). As expected, in experimental cheeses, the use of *Lactobacillus* strains as adjunct cultures significantly increased the population of lactobacilli as compared to the control. Moreover, lactobacilli increased their numbers until 60 days of ripening. Similar results were observed in studies involving the use of autochthonous LAB in cheese making (Piras et al., 2013; De Pasquale et al., 2019; Fusco et al., 2019). The counts of presumptive enterococci were higher in C3 cheese, where *E. faecalis* strain was present in the starter culture, with mean values ranging from 10^5^ to 10^7^ cfu/g, while in the other cheese types they showed mean concentrations from 10^2^ cfu/g in curd to about 10^5^ cfu/g at the end of ripening, in agreement with other studies (Giraffa, 2003; Piras et al., 2013; Fuka et al., 2017; Hanchi et al., 2018). With respect to yeast viable counts no marked differences were observed between the cheeses with the exception of C3, produced with an adjunct autochthonous probiotic *K. lactis* strain, that showed during manufacturing and ripening counts higher than both CC and C2 (produced without probiotic yeast) and C1 cheese, even though the latter was produced with the addition of the yeast *D. hansenii*. The yeast counts found in C3 cheese throughout maturation are in line or lower than those reported in others studies where yeast strains have been used as co-starter or adjunct in the production of cheeses (Lanciotti et al., 2005; De Freitas et al., 2008; Pereira Andrade et al., 2019). The similar yeast counts observed in control, C1 and C2 cheese could be related to the addition of the species *Geotrichum candidum* routinely used together with *Penicillium candidum* by the dairy plant as protective cultures in the cheese-making, in order to prevent or limit the growth of spoilage fungi in the cheese rind. To the best of our knowledge this is the first study reporting the use of autochthonous yeast strains with potential probiotic properties as an adjunct for the production of cheese.

**TABLE 3:** Viable counts (Log ufc/g) of presumptive lactococci, lactobacilli, enterococci and yeasts during manufacturing and ripening of control and experimental Caciotta cheeses.

**Cheese type**	**Days of ripening**				
	**Curd**	**1**	**15**	**30**	**60**
**Presumptive Lactococci**					
CC	8.05 ± 0.35^a^	8.30 ± 0.82^a^	7.75 ± 0.75^a^	8.55 ± 0.15^a^	8.17 ± 0.13^a^
C1	7.90 ± 0.58^a^	7.45 ± 1.56^a^	7.80 ± 1.50^a^	8.81 ± 0.11^a^	9.72 ± 0.38^a^
C2	9.39 ± 1.09^a^	8.89 ± 0.41^a^	9.20 ± 0.19^a^	9.09 ± 0.31^a^	9.05 ± 0.45^a^
C3	7.48 ± 1.12^a^	8.38 ± 0.38^a^	7.90 ± 0.93^a^	9.10 ± 0.10^a^	7.74 ± 1.04^a^
**Presumptive Lactobacilli**					
CC	1.50 ± 0.50^*b*^	2.65 ± 0.35^*a*^	4.15 ± 0.15^a^	5.54 ± 0.06^a^	6.16 ± 0.16^a^
C1	5.98 ± 0.98^ab^	7.42 ± 0.98^*b*^	8.50 ± 0.50^b^	8.61 ± 0.43^b^	9.15 ± 0.15^b^
C2	7.07 ± 0.77^a^	7.75 ± 1.15^b^	8.68 ± 0.68^b^	8.10 ± 0.85^b^	8.85 ± 0.35^b^
C3	7.09 ± 0.91^a^	7.75 ± 0.55^b^	8.78 ± 0.30^b^	8.93 ± 0.15^b^	8.93 ± 0.33^b^
**Presumptive Enterococci**					
CC	3.15 ± 1.15^a^	2.65 ± 0.65^a^	3.86 ± 0.74^a^	5.02 ± 0.18^a^	4.92 ± 0.67^a^
C1	2.75 ± 0.45^a^	2.75 ± 0.75^a^	4.15 ± 1.15^a^	5.42 ± 0.58^a^	5.3 ± 0.70^a^
C2	2.15 ± 0.15^a^	3.65 ± 0.15^a^	3.13 ± 1.06^a^	4.60 ± 2.00^a^	5.00 ± 2.00^a^
C3	5.65 ± 0.35^a^	6.58 ± 0.71^a^	6.14 ± 0.97^a^	7.24 ± 1.24^a^	7.74 ± 0.74^a^
**Yeasts**					
CC	3.50 ± 0.50^a^	3.16 ± 0.16^*a*^	3.79 ± 0.39^a^	4.23 ± 0.76^a^	3.65 ± 1.35^a^
C1	3.35 ± 0.65^a^	2.89 ± 0.19^a^	2.34 ± 1.00^a^	3.98 ± 0.80^a^	3.63 ± 0.37^a^
C2	3.15 ± 0.15^a^	3.04 ± 0.04^*a*^	3.28 ± 0.24^a^	4.99 ± 0.88^a^	3.60 ± 0.40^a^
C3	5.39 ± 0.69^a^	5.34 ± 0.56^*b*^	4.35 ± 0.35^a^	6.16 ± 0.82^a^	5.04 ± 0.56^a^

*Values are the mean ± standard error of triplicate samples of two batches of the same cheese type. Different superscripts in the same column indicate significant differences (*P* < 0.05).*

The colonies counted as presumptive lactococci, lactobacilli, enterococci and yeasts were randomly selected and identified by biochemical and molecular means, confirming their belonging to the inoculated species, the exception being *D. hansenii* that was not recovered in any of C1 samples and the presence of *E. faecalis* in CC and *E. faecium* in the other cheese types (data not shown). In general, the isolation of a varied microbiota in cheese is not unexpected since cheeses and related dairy environment (such as equipment, tanks, ripening rooms) are characterized by a complex microbiota that inevitably interact with the bacteria from milk and starter culture during cheese production (Montel et al., 2014).

**FIGURE 1:**
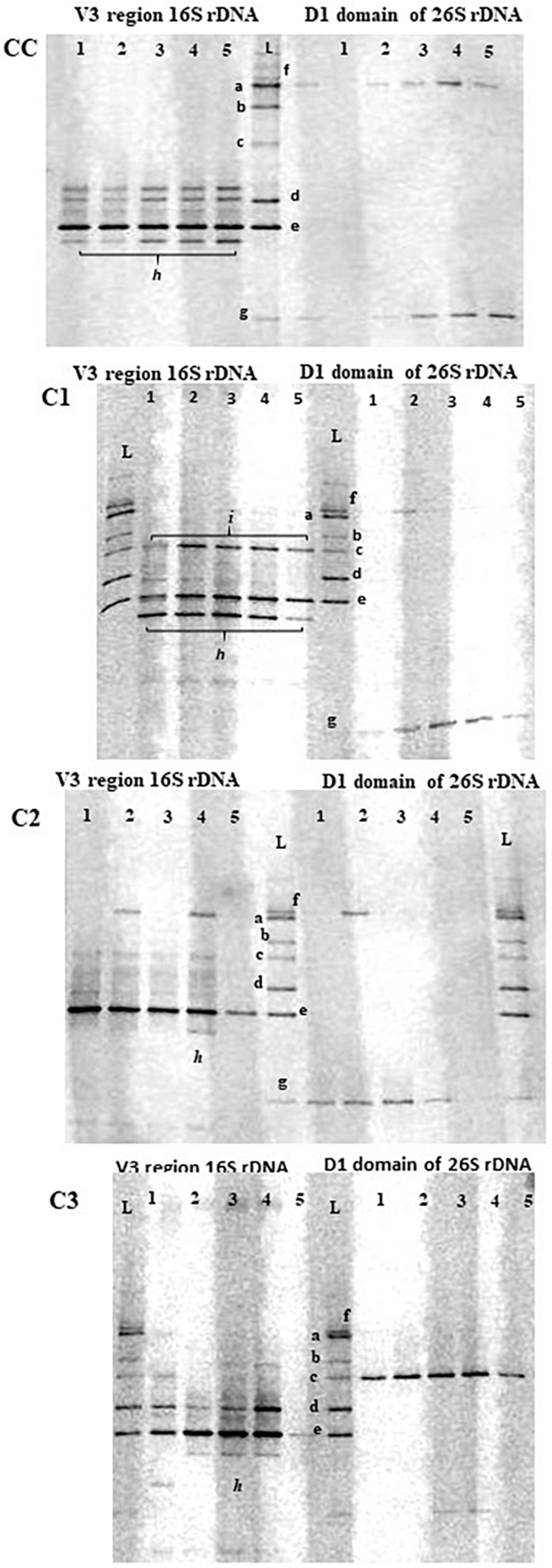
PCR-DGGE profiles of V3 16S and 26S rDNA regions in control (CC) and experimental (C1, C2, C3) cheese samples at different times of ripening. Lane 1, curd; lane 2, 1d-cheese; lane 3, 15d-cheese; lane 4, 30d-cheese; lane 5, 60d-cheese; L, identification ladder. a = *L. plantarum* ATCC 20174; b = *Debaryomyces hansenii* ATCC36239; c = *Kluyveromyces lactis* ATCC 56498; d = *E. faecalis* ATCC 29212; e = *L. lactis* ATCC 19435; f = *Geotrichum candidum*; g = *Penicillium candidum*; *h = S. thermophilus*; *i = E. faecium*.

In addition to the cultural analyses, a culture-independent approach using PCR-DGGE was used to monitor the presence of probiotic adjunct strains using the identification ladder indicated in MM (Figure 1). Although this molecular approach is not able to provide a complete picture of diversity and community composition of microbial ecosystems as that given by high-throughput sequencing methods, it can still be considered a helpful technique to monitor dominant LAB community changes in environmental and intestinal ecosystem (Yunita and Dodd, 2018).

**FIGURE 2:**
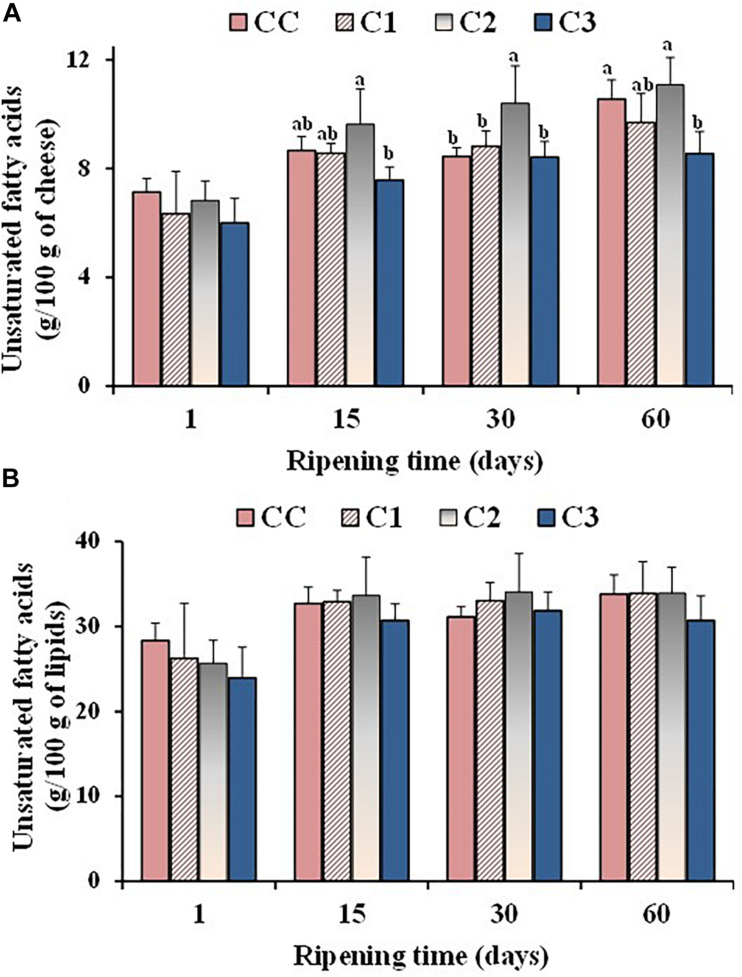
Values of total unsaturated fatty acids, expressed as g/100 g of cheese **(A)** and g/100 g of total lipids **(B)**, measured in the control (CC) and probiotic cheeses (C1, C2, C3) at different ripening times; (*n* = 6). ^*a,b,c*^ Samples with different superscript letters, within the same ripening time, are significantly different at *P* < 0.05 (One-way ANOVA).

In general, a good concordance was observed between PCR-DGGE and plate culture isolation, although a few inconsistencies were seen, as reported by other studies combining culture-dependent and –independent analyses (Pangallo et al., 2014; Silvetti et al., 2017). DGGE profiles of cheese samples consisted of just few bands mainly corresponding to the species used as starter and adjunct cultures, and they revealed the dominance of a band corresponding to *L. lactis* species in all cheeses throughout ripening. A weaker band corresponding to *L. plantarum* species was observed in all experimental probiotic cheeses. As the presence of *L. plantarum* probiotic strains during cheese making and maturation of probiotic cheeses was confirmed by cultural analysis and genotypic characterization by rep-PCR, the detection of weak bands in DGGE analysis could be due to different affinity of the primers to template DNA for different species, as observed by other authors (Bae et al., 2006; Mayrhofer et al., 2014). In additions to *L. lactis* and *L. plantarum*, other bands (indicated with the letter *h* and *i* in Figure 2) that could not be identified using our ladder were detected in all cheeses. These bands were excised and sequenced and were found to be homologous to *St. thermophilus* and *E. faecium* (*h* and *i*, respectively), typical adventitious LAB in cheese (Carafa et al., 2019).

DGGE analysis of 26S rDNA was used to investigate the presence of adjunct probiotic yeast strains *D. hansenii* and *Kl. lactis*. While *Kl. lactis* could be clearly detected in C3 cheeses at each stage of ripening, the band corresponding to *D. hansenii* was not seen in the DGGE gel of C1 cheeses, confirming the result of culture analysis. Since a detection limit of 10^3^ cfu/mL for DGGE analysis has been reported (Cocolin et al., 2000) and our cultural method has a detection limit of 10^2^ cfu/mL, on the basis of the metabolome results (see section “Metabolic Profile of Caciotta Cheeses During Maturation”) we have speculated that *D. hansenii* may be present in the cheese in a viable but not detectable state. DGGE analysis of 26S rDNA also showed a constant presence of the species *Geotrichum candidum* and *Penicillium candidum* in all cheeses throughout ripening.

### Sensory Analysis

A sensory evaluation of probiotic and control caciotta cheeses at 60 days of ripening was performed by six panelists. Mean ratings of descriptive attributes such as odor, aroma, basic tastes, texture and overall acceptability are documented in Table 4 and Supplementary Figure 2 in Supplementary Material. Significantly higher scores for aroma intensity were recorded in C3 cheeses with respect to C1 and C2 types, while C2 was perceived as the most bitter and acid with respect to the other groups. Furthermore, the textural parameters elasticity and solubility were significantly higher in CC and C1 cheeses. In general, the sensory profile of C1 cheese, found to be the most similar to CC cheese, and the highest scores for aroma obtained from C3 cheese may explain their slightly higher values for overall acceptability. Our results are in agreement with other studies investigating the sensory properties of Caciotta cheese produced with the addition of autochthonous LAB (Fusco et al., 2019; Bancalari et al., 2020).

**TABLE 4:** Sensory descriptors scores obtained for the control and probiotic Caciotta cheeses. Attributes were scored on a 7-point scale.

**Cheese type**			**Basic taste**					**Texture attributes**				
	**Appearance**	**Odor**	**Aroma**	**Acid**	**Sweetness**	**Bitter**	**Saltiness**	**Elasticity**	**Firmness**	**Solubility**	**Granularity**	**Overall**
												**acceptability**
CC	5.42 ± 0.38^a^	5.08 ± 0.49^a^	5.75 ± 0.27*^ab^*	2.75 ± 0.68^a^	4.42 ± 0.80^a^	2.33 ± 0.75^a^	4.00 ± 0.63^a^	4.50 ± 0.32^a^	2.42 ± 0.58^ab^	5.08 ± 0.49^a^	2.58 ± 0.58^ab^	4.42 ± 0.66^a^
C1	5.00 ± 0.45^a^	5.00 ± 0.77^a^	4.83 ± 0.60^b^	3.17 ± 0.68*^ac^*	3.25 ± 0.52^ac^	2.25 ± 0.27^a^	3.75 ± 0.70^a^	4.50 ± 0.45^a^	3.33 ± 0.52^a^	5.08 ± 0.38^a^	1.92 ± 0.66^a^	5.17 ± 0.41^a^
C2	3.42 ± 0.38^b^	4.08 ± 0.58^a^	4.75 ± 0.52^b^	4.33 ± 0.70*^bc^*	2.17 ± 0.26^bc^	4.08 ± 0.49^b^	4.33 ± 0.45^a^	3.33 ± 0.26^b^	2.67 ± 0.52^ab^	4.08 ± 0.20^b^	3.08 ± 0.80^b^	4.16 ± 0.81^a^
C3	4.83 ± 0.26*^ab^*	4.08 ± 0.49^a^	6.25 ± 0.42^a^	4.08 ± 0.50*^bc^*	2.92 ± 0.38^ac^	3.67 ± 0.41^ab^	4.50 ± 0.45^a^	3.42 ± 0.20^b^	2.00 ± 0.45^b^	4.08 ± 0.49^b^	2.17 ± 0.26*^ab^*	4.75 ± 0.70^a^

*The scores are expressed as mean values ± SD of two batches. Different letters within a column indicate significant differences (*P* < 0.05).*

### Fatty Acid Profile of Caciotta Cheese During Ripening

Quali-quantitative information on the main saturated (SFA) and unsaturated (UFA) FA that compose the lipid classes of Caciotta cheeses was obtained by HPLC analyses with ELSD detection. HPLC-ELSD is an alternative technology to UV analysis and is often employed when compounds lack sufficient absorptivity (like saturated FA). The ELSD detector quantifies any solute less volatile than the solvents and functional groups, FA chain-length, or saturation has little or no effect on the detector response (Aradottir and Olsson, 2005). However, a non-linear ELSD response for FA and a low repeatability of ELSD response factors have been reported (Cebolla et al., 1997; Aradottir and Olsson, 2005). Therefore, for the comparative assessment of the total FA profiles of Caciotta cheese samples, FA quantification was based on the internal normalization method (Amaral et al., 2004; Rosa et al., 2017), assuming that the detector response was the same for all FA. It was based on the received peak area, the results were normalized without correction factor and the FA contents were expressed as area percentages (% area). This HPLC–ELSD analysis was set up to analyze up to 16 FA (saturated + unsaturated FA) in one single analysis without any derivatization. The ELSD is suitable for the analysis of only C_12_–C_22_ FA since those with shorter chain length are not detected being too volatile (Bravi et al., 2006). FA compositions (expressed as % of total fatty acids) in control (CC) and probiotic Caciotta cheeses (C1, C2, C3) at 1 day of production measured by HPLC-ELSD are reported in Table 5. Control Caciotta cheese showed a concentration of approximately 53.3% of SFA (mainly palmitic acid 16:0, and stearic acid 18:0, 30, and 13%, respectively), 41.6% of monounsaturated FA (MUFA, mainly oleic acid 18:1 n-9 and 18:1 *trans* isomers, 35.6 and 5.9%, respectively), and 4.6% of polyunsaturated FA (PUFA), mainly constituted by conjugated linoleic acid (CLA) isomers and linoleic acid 18:2 n-6, 2.5 and 1.3%, respectively. The probiotic cheeses C1-C3 showed a SFA and UFA profile similar to the that of control cheese, with values of SFA, MUFA and PUFA in the range 53–55%, 40–41% and 4.6–5%, respectively. As indicated in Table 5, the absolute content of the main UFA detected in control and probiotic Caciotta cheeses at 1 day of production by HPLC with DAD detection and values, expressed as g/100 g of Caciotta cheese on a wet basis, showed UFA amounts of experimental cheeses in line with those measured in the control. The average values for the main UFA of both control and probiotic cheeses were in the range: 3.5–4.2 g/100 g of cheese for 18:1 n-9, 1.3-1.5 g/100 g for 18:1 *trans* isomers, 0.4-0.5 g/100 g for 18:2 n-6, and 0.3-0.4 g/100 g for CLA isomers.

**TABLE 5:** Fatty acid profile of control (CC) and probiotic Caciotta cheese (C1, C2, C3) at 1 d of production measured by HPLC analysis with ELSD and DAD detection.

	**% Area (ELSD detection)**				**g/100 g of Caciotta (DAD detection)**			
	**CC**	**C1**	**C2**	**C3**	**CC**	**C1**	**C2**	**C3**
12:0	0.10 ± 0.02	0.10 ± 0.05	0.13 ± 0.06	0.11 ± 0.07	−	−	−	−
14:0	9.93 ± 0.78	10.31 ± 0.53	10.83 ± 1.19	11.20 ± 0.68	−	−	−	−
14:1	−	−	−	−	0.04 ± 0.00	0.03 ± 0.00	0.03 ± 0.00	0.03 ± 0.00
16:0	30.50 ± 1.36	31.89 ± 2.15	30.23 ± 2.57	30.14 ± 2.30	−	−	−	−
16:1 n-7	0.15 ± 0.02	0.17 ± 0.03	0.18 ± 0.03	0.19 ± 0.01	0.14 ± 0.01	0.12 ± 0.02	0.13 ± 0.01	0.12 ± 0.01
18:0	12.80 ± 0.89	12.44 ± 1.14	12.04 ± 0.96	12.52 ± 1.23	−	−	−	−
18:1 *trans* isomers	5.87 ± 0.65	5.71 ± 0.45	5.85 ± 0.59	5.91 ± 0.77	1.50 ± 0.22	1.36 ± 0.46	1.45 ± 0.27	1.26 ± 0.27
18:1 n-9	35.58 ± 2.49	34.30 ± 1.39	35.15 ± 2.45	34.43 ± 3.03	4.16 ± 0.26	3.69 ± 0.85	3.98 ± 0.37	3.52 ± 0.49
18:2 n-6	1.28 ± 0.15	1.23 ± 0.07	1.31 ± 0.15	1.30 ± 0.01	0.52 ± 0.03	0.45 ± 0.11	0.49 ± 0.05	0.43 ± 0.06
CLA isomers	2.50 ± 0.04	2.50 ± 0.22	2.76 ± 0.18	2.72 ± 0.33	0.41 ± 0.04	0.35 ± 0.04	0.38 ± 0.03	0.34 ± 0.01
18:3 n-3	0.56 ± 0.10	0.58 ± 0.07	0.62 ± 0.16	0.55 ± 0.04	0.27 ± 0.04	0.24 ± 0.08	0.26 ± 0.05	0.22 ± 0.04
18:3 n-6	−	−	−	−	0.01 ± 0.00	0.01 ± 0.00	0.01 ± 0.00	0.01 ± 0.00
18:4 n-3	−	−	−	−	trace	trace	trace	trace
20:4 n-6	0.30 ± 0.02	0.32 ± 0.02	0.34 ± 0.06	0.35 ± 0.02	0.06 ± 0.00	0.05 ± 0.01	0.05 ± 0.00	0.05 ± 0.01
20:5 n-3	−	−	−	−	0.02 ± 0.00	0.02 ± 0.00	0.02 ± 0.00	0.02 ± 0.00
22:6 n-3	−	−	−	−	0.01 ± 0.00	0.01 ± 0.00	0.01 ± 0.00	0.01 ± 0.00
SFA	53.33 ± 2.43	54.74 ± 1.38	53.24 ± 2.68	53.982.42	−	−	−	−
MUFA	41.60 ± 2.32	40.18 ± 1.37	41.18 ± 2.55	40.53 ± 2.62	5.83 ± 0.47	5.20 ± 1.33	5.59 ± 0.63	4.93 ± 0.77
PUFA	4.65 ± 0.22	4.63 ± 0.23	5.04 ± 0.37	4.92 ± 0.29	1.35 ± 0.05	1.18 ± 0.25	1.28 ± 0.10	1.25 ± 0.14
Others	0.43 ± 0.07	0.45 ± 0.13	0.55 ± 0.13	0.57 ± 0.09	−	−	−	−

*SFA, saturated fatty acids; MUFA, monounsaturated fatty acids; PUFA, polyunsaturated fatty acids. Data reported are the mean ± standard deviation (SD) of 6 values (*n* = 6).*

A similar trend and no significant differences were measured in the SFA, MUFA and PUFA % levels among the different batches of Caciotta cheeses at all ripening stages (Supplementary Figure 3 in Supplementary Material).

Some differences were observed in the absolute amounts of UFA during ripening. Figure 2A shows the total UFA amounts (expressed as g/100 g of cheese on a wet basis) measured in the control and probiotic cheeses at different ripening times. The absolute values of UFA increased during ripening in all cheese types mostly due to a corresponding decrease in the moisture content, as indicated by the high negative correlation coefficients measured between UFA absolute values/moisture level during ripening for CC (r = −0.9736), C1 (r = −0.9661), C2 (r = −0.8365), and C3 (r = −0.8480). Significant differences were detected in the UFA absolute values among cheese types from 15 days of ripening, with sample C2 and C3 showing the highest and the lowest UFA contents, respectively. These differences were ascribable to a different moisture level in Caciotta cheeses. In fact, no significant differences were observed between experimental and control group at the different ripening times when total UFA levels were measured as g per 100 g of lipids (Figure 2B), suggesting that the addition of probiotic bacteria did not affect the composition of FA that make up triglycerides of cheese fat. Similar results were reported by Perotti et al. (2014), who did not observe modifications in the FA composition (expressed as %, g FA/100 g of total FA) of Argentinean semi-hard ovine cheese made with a mix of probiotic microorganisms.

**FIGURE 3:**
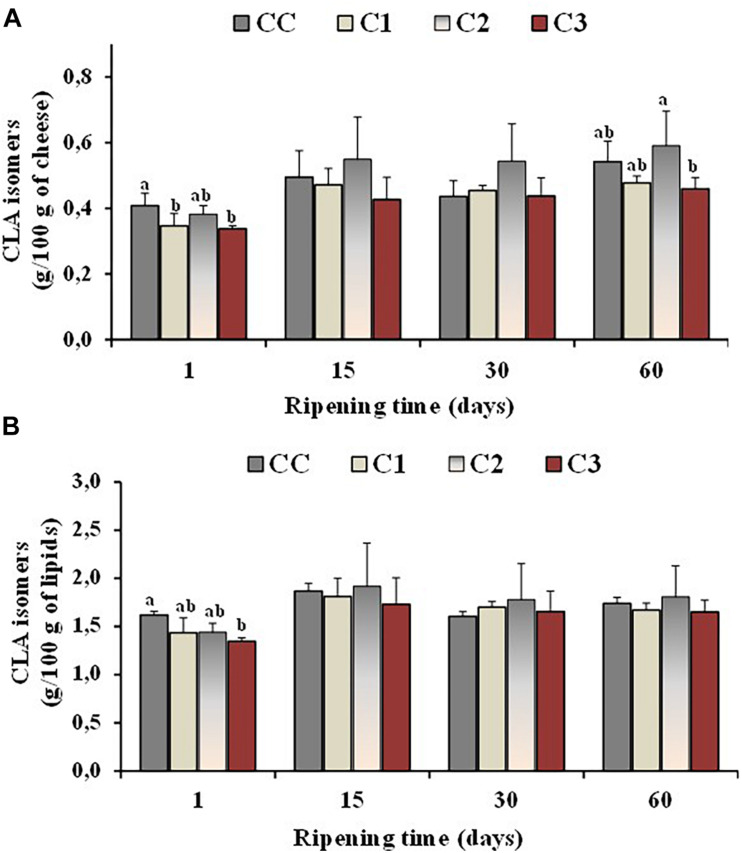
Total values of CLA isomers, expressed as g/100 g of cheese **(A)** and g/100 g of total lipids **(B)**, measured in the control (CC) and probiotic cheeses (C1, C2, C3) at different ripening times; (*n* = 6). ^*a,b,c*^ Samples with different superscript letters, within the same ripening time, are significantly different at *P* < 0.05 (One-way ANOVA).

Isomers of conjugated linoleic acid (or CLAs) are of particular interest from a nutritional point of view, being *cis*-9, *trans* -11 CLA (rumenic acid) the most representative. Experimental evidence has suggested that CLA may have antiatherosclerotic, anticarcinogenic, antidiabetic and immunomodulanting effects (Mele et al., 2011; Prandini et al., 2011). Since CLA is naturally present in milk from ruminants, cheese represents a good source of this FA, its content in cheese being usually related to the CLA level of the unprocessed milk (Perotti et al., 2014). Sheep cheeses reported a higher level of CLA with respect to cow and goat cheeses (Prandini et al., 2011). Additional CLA quantities could be produced during manufacturing and ripening of cheeses, as some cheese-related microorganisms including probiotic bacteria have shown the ability to produce CLA (Laskaridis et al., 2013; Perotti et al., 2014). Figure 3 shows the total values of CLA isomers, expressed as g/100 g cheese (Figure 3A) and g/100 g lipids (Figure 3B), measured in the control and probiotic cheeses C1-C3 at different ripening times. In our study, the absolute values of CLA isomers, like all the others FA, increased per 100/g of cheese in control and probiotic cheeses with ripening time, and the highest values were measured at 60 days ripening in the cheese samples characterized by a lower rate of moisture (CC and C2). Similar CLA contents were measured per 100 g of lipids, among Caciotta cheese batches at all ripening stages indicating that the probiotic cultures did not affect the CLA content of cheeses with respect to the control. Similar results were reported by other authors for ovine, caprine and bovine cheeses (Gursoy et al., 2012; Perotti et al., 2014).

### Total Cholesterol and α-Tocopherol Levels of Caciotta Cheese During Ripening

**FIGURE 4:**
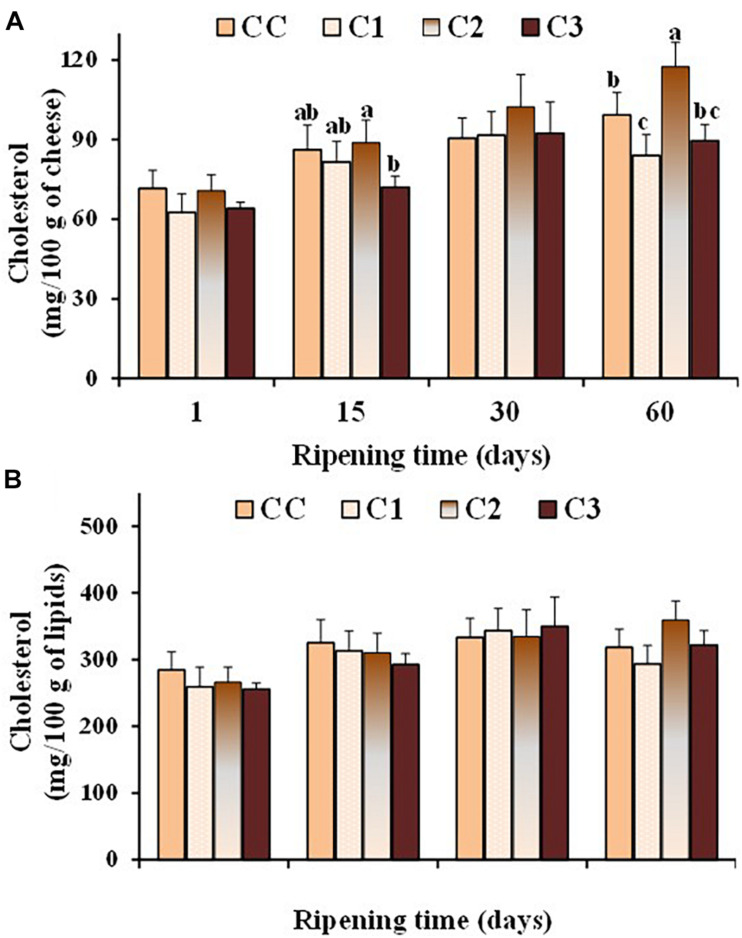
Values of total cholesterol, expressed as mg/100 g of cheese **(A)** and mg/100 g of total lipids **(B)**, measured in the control (CC) and probiotic cheeses (C1, C2, C3) at different ripening times; (*n* = 6). ^*a,b,c,d*^ Samples with different superscript letters, within the same ripening time, are significantly different at *P* < 0.05 (One-way ANOVA).

Dairy products play a major role in supplying dietary cholesterol, since cholesterol is the predominant sterol of milk (> 95 g/100 g of total sterol) (Collins et al., 2003). The association between plasma cholesterol and atherosclerosis has emphasized the importance of the assessment of cholesterol in dairy products (Marrone et al., 2014). Several probiotic strains have been shown to possess *in vitro* cholesterol-lowering ability (Ooi and Liong, 2010). Figure 4 shows the values of total cholesterol, expressed as mg/100 g cheese and mg/100 g lipids (Figures 4A,B, respectively), measured in the control and probiotic cheeses at different ripening times. As expected, cholesterol content (mg/100 g cheese) increased in all cheese types during ripening because of the loss of water. After 60 days of ripening, the total cholesterol content was in the range 84–117 mg/100 g of cheese, with lower values observed in the probiotic cheeses C1 and C3, with respect to control CC and C2 sample. No significant differences were found in the cholesterol content expressed as mg/100 g of fat between control and probiotic cheeses even though in-vitro cholesterol-lowering *Lactobacillus* strains were used in C1 e C3 cheeses. On the other hand, possible differences between the expression of functional properties *in vitro* and *in vivo* have been pointed out, as various factors related to the environmental conditions and/or interaction with microbial communities prevailing *in situ* could affect the efficiency of the strains. In the study by Albano et al. (2018) seven LAB strains showed a lower ability to reduce cholesterol in cheese than in broth. Our results highlight the importance to perform in situ studies to confirm the in vitro functional characteristics of probiotic strains in order to assess their health-promoting properties and their performance as novel probiotic.

**FIGURE 5:**
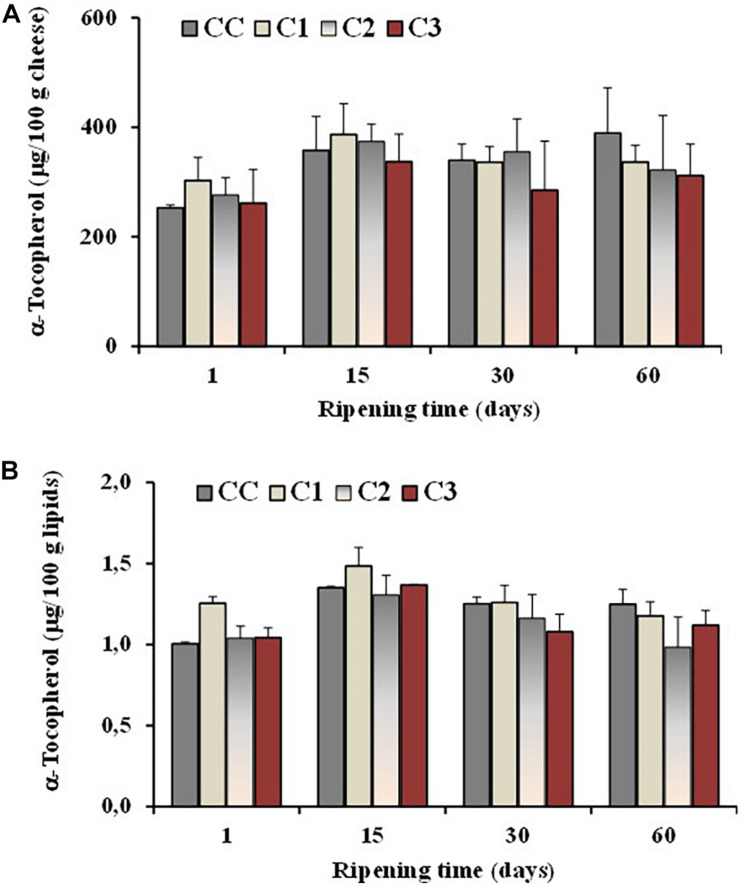
Total values of α-tocopherol, expressed as μg/100 g of cheese **(A)** and μg/100 g of total lipids **(B)**, measured in the control (CC) and probiotic cheeses (C1, C2, C3) at different ripening times; (*n* = 6).

The level of the antioxidant α-tocopherol was also determined in all cheeses during ripening. In general, cheese is considered a product stable to oxidation (Mele et al., 2011), characterized by the presence of antioxidants like vitamin E, vitamin A and β-carotene (Revilla et al., 2014). Vitamin E is known to act primarily as a liposoluble antioxidant, protecting PUFA and related substances from peroxidation, and α-tocopherol is the main component with vitamin E activity in sheep milk and cheese (Revilla et al., 2014). The effect of different probiotic organisms and ripening time on the α-tocopherol content of the control and probiotic cheeses is presented in Figure 5. A little increase in concentration, expressed as μg/100 g cheese, was observed in all cheese batches at 15 days of ripening, followed by similar or lower amount at 30 and 60 days of maturation (Figure 5A). No significant differences were observed among control and experimental cheeses and the average values for α-tocopherol of both control and probiotic cheeses were in the range 312–389 μg/100 g of cheese and 1–1.2 μg/100 g of lipids after 60 days of ripening (Figure 5B).

### Metabolic Profile of Caciotta Cheeses During Maturation

Metabolomics analysis of cheese has been shown to be a valuable tool to investigate the association between the biochemical events occurring in this food and maturation process (Piras et al., 2013), microbial culture-dependent methods (Pisano et al., 2016), sensory properties (Ochi et al., 2012a), or cheese quality (Mazzei and Piccolo, 2012). In the present study, we have performed a NMR-based metabolomics analysis of the aqueous extract of Caciotta cheese to evaluate and compare the changes of the metabolic profile due to the ripening time and the added cultures.

**FIGURE 6:**
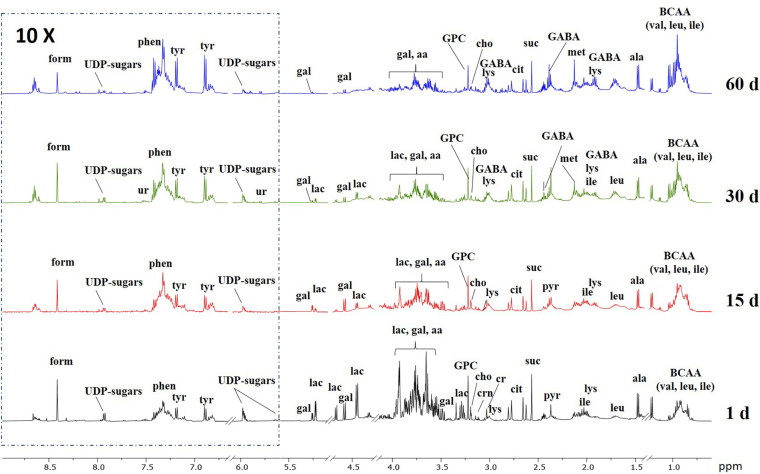
Representative ^1^H NMR spectra of the aqueous extracts of control Caciotta cheese at different ripening days. Abbreviations: aa, amino acids; ala, alanine; cit, citric acid; cho, choline; cr, creatine; crn, creatinine; form, formic acid; GABA, γ-aminobutyric acid; gal, galactose; GPC, glycero-phosphocholine; ile; isoleucine; lac, lactose; leu, leucine; lys, lysine; met, methionine; phen, phenylalanine; pyr, pyruvic acid; suc, succinic acid; tyr, tyrosine; UDP-sugars, uridine diphosphate-related sugars; ur, uracile; val, valine.

Representative ^1^H NMR spectra of the aqueous extracts of control Caciotta at different ripening days are reported in Figure 6, while those of C1, C2 and C3 cheeses are depicted in the Supplementary Material (Supplementary Figures 4–7). A variety of amino acids, carbohydrates, osmolytes, and organic acids were detected. In good agreement with the literature (Consonni and Cagliani, 2008; Rodrigues et al., 2011; Piras et al., 2013), it can be visually observed that ripening time has an impact on the NMR profile of all cheeses, as pointed out, in particular, by the increasing temporal intensities of many amino acid peaks and the decreasing of lactose signals. Among the various NMR peaks, those from lactic acid (La) were predominant in all Caciotta aqueous extracts at all ripening times (Supplementary Figure 4 in the Supplementary Material). A high content of La is typical of the initial stage of cheese when the starter cultures cause a slight and rapid acidification of milk. The occurrence of high levels of La over the maturation time of Caciotta, indicated a sustained microbial activity during the ripening period. Compared to control, the levels of La in the experimental cheeses produced with autochthonous strains increased more markedly after 15 days of maturation, particularly in C1 and C3 (Supplementary Figure 8 in Supplementary Material), then decreased up to 60 days of ripening, reaching the lowest values in C3, presumably as a consequence of the lactate metabolism of the *K. lactis* strain included in the culture (Fadda et al., 2004). Since lactic acid is the most abundant organic acid in Caciotta, its content changes are reasonably the main source of the observed pH variations (Table 2).

**FIGURE 7:**
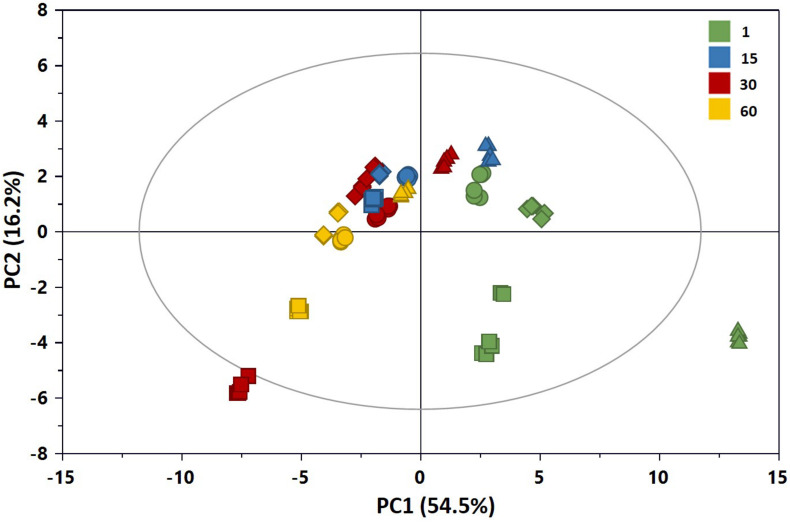
PC1 vs. PC2 scores plot of the PCA model built with the ^1^H NMR spectra of the aqueous extracts of Caciotta (*R*^2^X = 0.707; *Q*^2^ = 0.698): CC (•), C1 (▲), C2 (◆), C3 (■). The color of scores denotes the ripening days. *The reader is remanded to the color version of the figure*.

A preliminary multivariate statistical analysis of the whole NMR data set was performed by PCA in order to explore the intrinsic variability. Due to the high intensity of lactic acid peaks, the corresponding bins were removed prior to analysis in order to monitor possible changes of the metabolic profiles due to the less abundant metabolites. Figure 7 shows the PCA scores plot built with the first two principal components (PCs), PC1 and PC2 explaining 54.5 and 16.2% of the total variance, respectively. All samples in the plots were within the 95% Hotelling’s T-squared ellipse, except those of C1 at 1 day of ripening, characterized by higher levels of lactose compared to the other samples. As it can be observed by the score distribution for each time point, we obtained a good reproducibility of NMR data in terms of replicates and trials. Furthermore, the score movement from the right to the left indicated a continuous metabolic change during cheese maturation. Differences in the scores distribution were visible also in terms of added cultures, in particular at the beginning of preparation. Overall, these findings suggested variations in the metabolome of cheeses in relation to both ripening time and added culture.

To focus on the metabolic changes mostly correlated with the ripening time, an OPLS model was built for each Caciotta cheese type (Supplementary Figure 9 in Supplementary Material). All models were characterised by high statistical values, yielding a *Q*^2^ ≥ 0.95. Each model was further validated by permutation tests (*n* = 400), providing the *Q*^2^ and *R*^2^ values of the permutated models lower than the original ones (data not shown), and by CV-ANOVA (*p* < 0.0001) to ensure that there was no overfitting. The analysis of the S-line correlation coefficient plots evidenced for all cheeses a general increase of the content of free amino acids (AA) and organic acids during ripening, and a concomitant decrease of the carbohydrate levels. Among these metabolites, only a few exhibited a statistically significant correlation with cheese aging (*p*(cov) ≥|0.05|; *p*(cor) ≥|0.5|). In particular, branched chain amino acids (BCAA: valine, leucine, isoleucine) were positively correlated with time in all cheeses. Additionally, a positive correlation was observed for methionine, leucine, and 4-aminobutyric (GABA) only in CC and C3, for succinic acid only in C1 and C2, for alanine only in C3 and for acetic acid only in C2. Finally, for all cheeses, the most significant negative correlation with time was observed for lactose.

**FIGURE 8:**
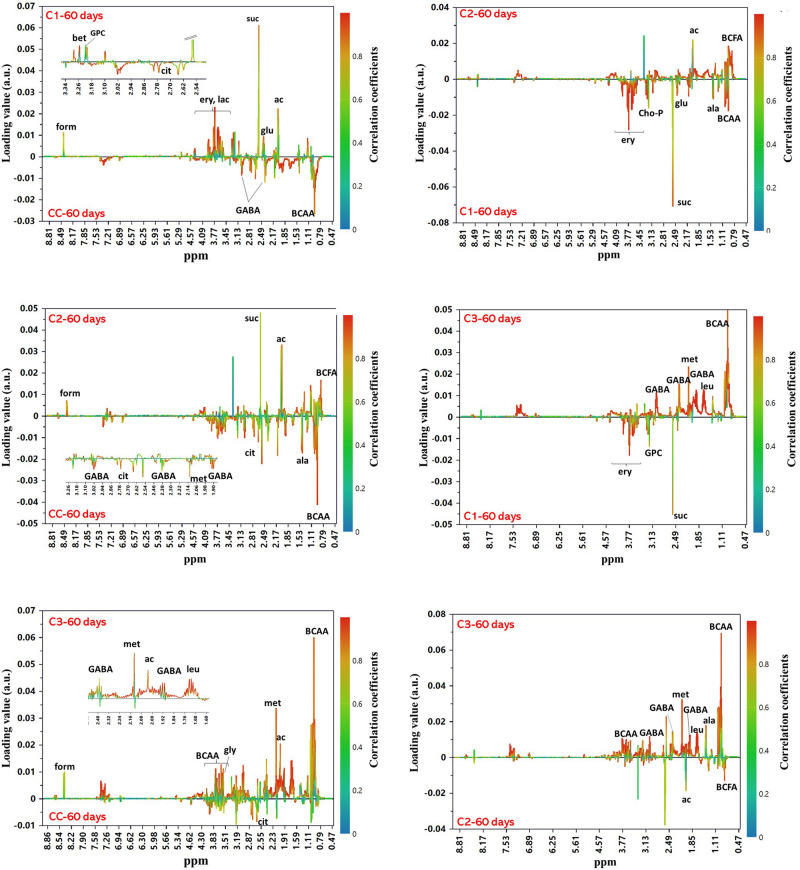
OPLS-DA S-line correlation coefficient plots for the pairwise comparisons between groups of cheeses at 60 days of ripening. For all models *Q*^2^Y > 0.95 and CV-ANOVA *p*-value < 0.0001. Only the signals from the major contributing metabolites toward the class separation are labeled (cutoff values: *p*(cov) ≥|0.05| and *p*(cor) ≥|0.5|). Abbreviations: ac, acetic acid; ala, alanine; BCAA, branched-chain amino acids; BCFA, branched-chain fatty acids; cho-P, phospho-choline; cit, citric acid; ery, erythritol; form, formic acid; GABA, γ-aminobutyric acid; gly, glycine; glu, glutamate; GPC, glycero-phosphocholine; lac, lactose; leu, leucine; met, methionine; suc, succinic acid.

In order to examine the differences among the NMR profiles of cheeses in terms of the type of added culture, OPLS-DA models were built for pairwise comparisons among samples at 60 days of ripening. All models were found robust following permutation test (*n* = 400) and CV-ANOVA (*p* < 0.0001). The score plots of the models are shown in Supplementary Material (Supplementary Figure 10). Looking for candidate metabolites with an important impact on the group separation (Figure 8), we could note that C3 cheese had the highest content of BCAA followed in decreasing order by CC, C1, and C2. C3 exhibited also the highest levels of methionine and GABA, the latter being present only in the spectra of C3 and CC. As to the organic acids, CC was characterized by the highest content of citric acid, while high levels of formic, acetic and succinic acids were significantly associated with the use of autochthonous cultures. Acetic acid was more abundant in C2, while succinic acid in C1. In C1, of interest is the presence of erythritol. This polyol has been reported to occur in different type of cheeses by osmotolerant yeasts in response to salt stress (Breuer and Harms, 2006; Tomaszewska et al., 2014), and its presence may be indicative of metabolic activity by the *D. hansenii* strain added to C1, even though it was not detected either in culture or by DGGE analysis. Significantly higher levels of branched-chained fatty acids characterized C2 compared to the other cheeses.

Overall, the above-mentioned compositional changes observed in the metabolic profile of Caciotta relative to ripening time are in line with the occurrence of cheese fermentation (Mcsween, 2004). Lactose is the most abundant carbohydrate in milk. It is mostly consumed at the beginning of the fermentation as the main energy source for the growth of microorganisms. As fermentation progressed, the bacteria degraded also the milk proteins and released peptides and free AA, contributing to the general flavor and sensory quality of cheese. In particular, BCAA and methionine are some of the major precursors of cheese aroma compounds. These metabolites were particularly abundant in C3 and CC that, according to our sensorial analysis, were also the cheeses with the most intense aroma. GABA is the decarboxylation product of L-glutamate which is naturally present in caseins. It possesses several physiological activities, such as neurotransmission, antianxiety, and improves brain function and long-term memory (Wu and Sun, 2015). During fermentation, several LAB and NSLAB have been shown to produce GABA in response to stress due to acidic conditions (Dhakal et al., 2012; Valenzuela et al., 2019). This activity appears to vary widely among LAB strains and to depend on fermentation parameters such as pH and temperature. GABA has been also associated with a sour flavor note (Ochi et al., 2012b). Its high levels in C3 may contribute to the distinctive flavor profile of this cheese, besides giving also an added value compared to the other Caciotta due to its potentially beneficial health-promoting effects. Also organic acids have an important role on the sensory properties of cheese (Singh et al., 2003), besides acting as natural preservatives. They may arise from the hydrolysis of fatty acids, bacterial growth, or the addition of acidulants during cheesemaking. Succinic acid, in particular, is known to be produced by citrate-fermenting strains of LAB (Kaneuchi et al., 1988), while acetic and formic acids are derived mainly from lactose by the hetero-fermentative metabolism of NSLAB (McSweeney and Fox, 2004). The relatively lower levels of succinic and acetic acid and higher abundance of citric acid in the commercial Caciotta compared to C1, C2, and C3 suggests the presence of a higher proportion of non-citrate-fermenting strains in the control cheese.

## Conclusion

Cheese has been demonstrated to be an optimal carrier product to deliver living probiotic bacteria, and autochthonous potential probiotic strains would be the best choice for use as adjunct cultures since they should be well-adapted to this food environment (Castro et al., 2015; Fusco et al., 2019; Bancalari et al., 2020). The data obtained in this study have indicated the applicative potential of autochthonous LAB cultures, containing putative probiotic *Lactobacillus* and *Kluyveromyces* strains, for the production of ovine Caciotta cheese. The use of combined inoculums with autochthonous cultures and probiotic strains did not statistically affect the gross composition and lipid profile of experimental Caciotta with respect to the control, while improving the sensory characteristics in case of C1 and C3 cheeses. The NMR-based metabolomics approach used in our study highlighted differences in the cheese metabolome as a function of both ripening time and added autochthonous cultures. Both the *Kluyveromyces* and *Lactobacillus* probiotic strains survived manufacturing process and retained their viability till the end of ripening, the latter with a concentration much higher than 10^6^ cfu/g, the amount of probiotic bacteria suggested at the time of consumption in order to exert a positive effect on human health. Further studies will aim to assess the survival of the probiotic strains in the human intestinal tract of volunteers fed with probiotic Caciotta cheese and to investigate the influence of the probiotic cheese intake on human metabolomics profile.

## Data Availability Statement

The raw data supporting the conclusions of this article will be made available by the authors, without undue reservation.

## Ethics Statement

The panel test participants provided written informed consent to participate in this study. Sardinian Region ethic committee did not require the study to be reviewed or approved by an ethic committee.

## Author Contributions

AR and MP contributed to the experimental design of the study, interpretation of results, and wrote the manuscript. DP, VM, and SV carried out the experiments. FC performed NMR measurements and analyzed the data. MF carried out the DGGE experiments and supervised the study. SC contributed to the interpretation of the results and the critical revision of the manuscript. All authors read and approved the final manuscript.

## Conflict of Interest

The authors declare that the research was conducted in the absence of any commercial or financial relationships that could be construed as a potential conflict of interest.

## Footnotes

1 http://www.fdb.ca
