# Supplementary material for: Influence of Autochthonous Putative Probiotic Cultures on Microbiota, Lipid Components and Metabolome of Caciotta Cheese
Source: Front Microbiol. 2020 Oct 21;11:583745. doi: 10.3389/fmicb.2020.583745 (PMC7609418; doi:10.3389/fmicb.2020.583745)
Supplement: Supplementary file 1 [file Data_Sheet_1.pdf]

SUPPLEMENTARY MATERIAL

Influence of Autochthonous Putative Probiotic Cultures on Microbiota, Lipid Components and Metabolome of Caciotta Cheese

Maria Barbara Pisano[1], Antonella Rosa[2*], Danilo Putzu[2], Flaminia Cesare Marincola[3*], Valentina Mossa[1], Silvia Viale[1], Maria Elisabetta Fadda[1], Sofia Cosentino[1*]

[1]Department of Medical Sciences and Public Health, University of Cagliari, Italy
[2]Department of Biomedical Sciences, University of Cagliari, Italy
[3]Department of Chemical and Geological Sciences, University of Cagliari, Italy

*Corresponding Authors
Antonella Rosa: anrosa@unica.it
Flaminia Cesare Marincola: flaminia@unica.it
Sofia Cosentino: scosenti@unica.it

Table S1 - In vitro functional characteristics related to technological, probiotic and safety properties of the microbial strains used in the study.

Cheese type	Microbial strains	Technological properties	Safety properties	Probiotic properties	Reference
C1	*L. lactis* 6MRSLS5	• Casein hydrolysis +<br>• 6.5% NaCl +<br>• *B*-galactosidase +<br>• Nisin z producer	-	-	Pisano et al., 2015
	L. plantarum 19/20711	• Casein hydrolysis +<br>• Citrate utilization +	• No hemolysis in blood agar plates<br>• No decarboxylation of lysine, histidine, ornithine and tyrosine<br>• Sensitive to ampicillin, amoxicillin/clavulanic acid, tetracycline, erythromycin, rifampicin	• TDCA and GDCA hydrolysis +<br>• Raffinose fermentation +<br>• Antibacterial activity vs *S. aureus*, *L. monocytogenes*, *E. coli* O157:H7, Salmonella Typhymurium,<br>• Antifungal activity vs *Candida* spp.<br>• Able to survive to gastric and intestinal juice (survival 90%)<br>• Adhesion to Caco2-cells (7.3%)<br>• Cholesterol assimilation +*	Pisano et al., 2014<br>* Poster presentation at National Congress SItI 2017*
	D. hansenii (FS6DH1)	• Glucose fermentation +<br>• Lactate assimilation +<br>• Lipolytic activity +	–	• Growth in the presence of 0.3% bile acids<br>• Growth at pH 3.0<br>• Able to survive to gastric and intestinal juice (survival 85%)<br>• Adhesion to Caco2-cells (50%)	Master thesis (unpublished)
C2	*L. lactis* 1FS171M	• Casein hydrolysis +<br>• Milk coagulation +<br>• 6.5% NaCl +<br>• Good acidifyng activity	–	–	Cosentino et al., 2002
	L. plantarum 62LP39B	• Casein hydrolysis +<br>• Milk coagulation +<br>• Citrate utilization +	• No hemolysis in blood agar plates<br>• No decarboxylation of lysine, histidine, ornithine and tyrosine<br>• Sensitive to ampicillin, amoxicillin, piperacillin, gentamycin, tetracycline, erythromycin, clindamycin, rifampicin	• GDCA hydrolysis +<br>• Antibacterial activity vs *S. aureus*, *L. monocytogenes*, *E. coli* O157:H7, Salmonella Typhymurium.<br>• Antifungal activity vs *Candida* spp.<br>• Able to survive to gastric and intestinal juice (survival 93%)<br>• Adhesion to Caco2-cells (10%)	Pisano et al., 2008 (*L. plantarum* DBS273 renamed as *L. plantarum* 62LP39B)

C3	*E. faecalis* 3M17LS5	• Casein hydrolysis +<br>• Citrate utilization +<br>• Moderate acidifying activity<br>• Antibacterial activity vs *S. aureus, L. monocytogenes*	• No hemolysis in blood agar plates<br>• No decarboxylation of lysine, histidine, ornithine and tyrosine<br>• Virulence genes: *efa*A + agg +, *CylM* - , *gelE* -<br>• Sensitive to ampicillin, amoxicillin, vancomycin tetracycline,	–	Cosentino et al., 2004
	L. plantarum 11/20966	• Casein hydrolysis +<br>• Milk coagulation +<br>• Citrate utilization +	• No hemolysis in blood agar plates<br>• No decarboxylation of lysine, histidine, ornithine and tyrosine<br>• Sensitive to ampicillin, amoxicillin/clavulanic acid, tetracycline, erythromycin, rifampicin	• TDCA and GDCA hydrolysis +<br>• Able to survive to gastric and intestinal juice (survival 90%)<br>• Adhesion to Caco2-cells (11%)<br>• Antibacterial activity vs *S. aureus*, *L. monocytogenes*, E. coli O157:H7, *Salmonella* Typhymurium,<br>• Antifungal activity vs Candida spp.<br>• Cholesterol assimilation +*	Pisano et al., 2014<br>* Poster presentation at National Congress SItI 2017*
	K. lactis 17KL2	• Casein hydrolysis +<br>• Moderate acidifying activity<br>• Glucose and lactose fermentation +<br>• Lactate assimilation +<br>• Killer activity	• No hemolysis in blood agar plates<br>• Sensitive to the clinical antimycotic Itraconazole, Voriconazole, Amphotericin B, Ketoconazole, Fluconazole	• TDCA and GDCA hydrolysis +<br>• Able to survive to gastric and intestinal juice (survival 90%)<br>• Adhesion to Caco2-cells (68%)<br>• Antibacterial activity vs *S. aureus*, *E. coli* O157:H7, *Salmonella* Enteritidis	Fadda et al., 2004<br>Fadda et a., 2017

*Pisano M. B., Deiana M., Deplano M., Fadda M.E., Viale S., Mossa V., Cosentino S. Assimilazione del colesterolo e produzione di CLA in batteri lattici di origine casearia (Cholesterol assimilation and CLA production by lactic acid bacteria of dairy origin). 50° Congresso Nationale SItI Torino, 2017.

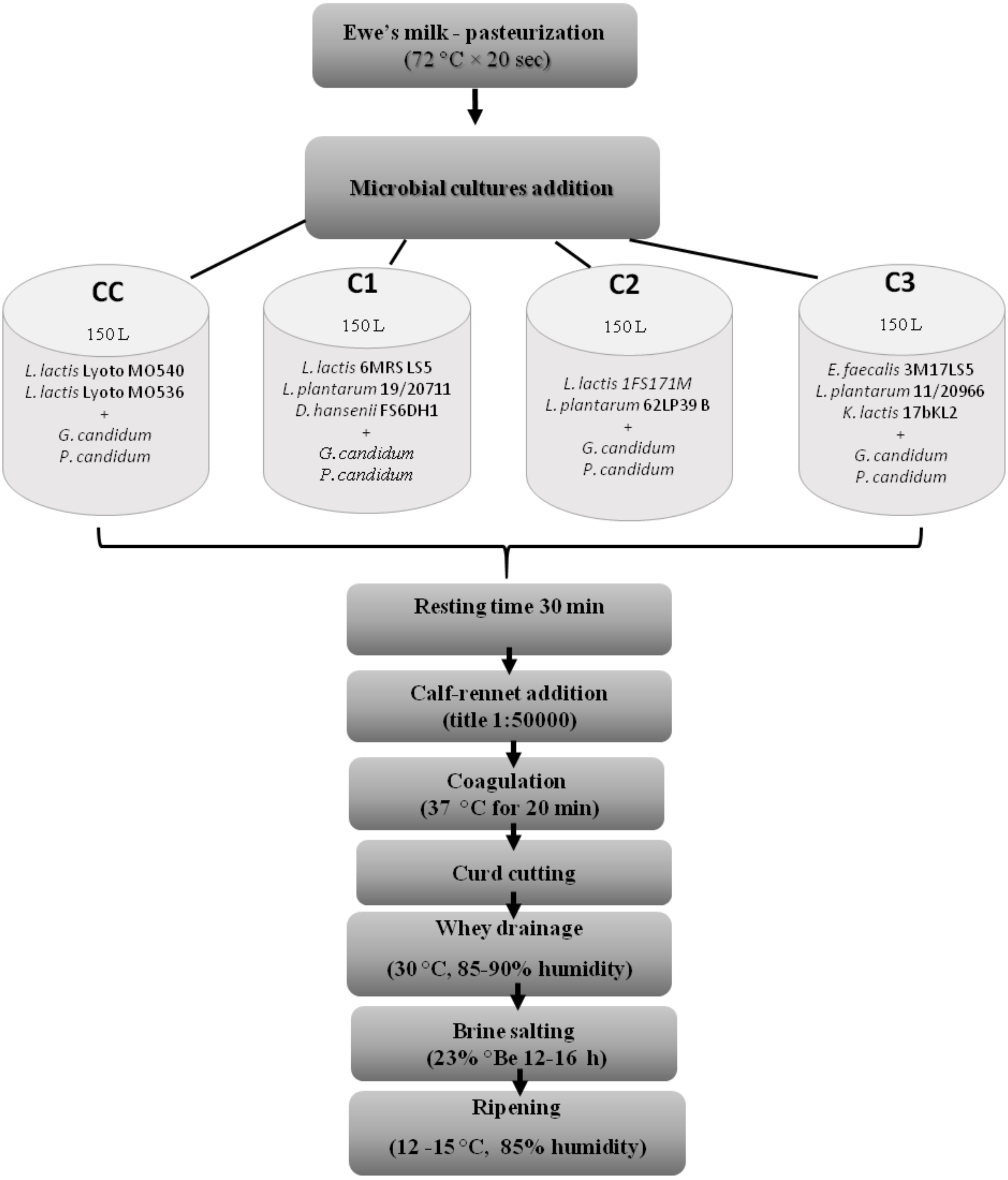

Figure S1. Flow diagram of the manufacturing procedure for control and experimental probiotic cheeses.

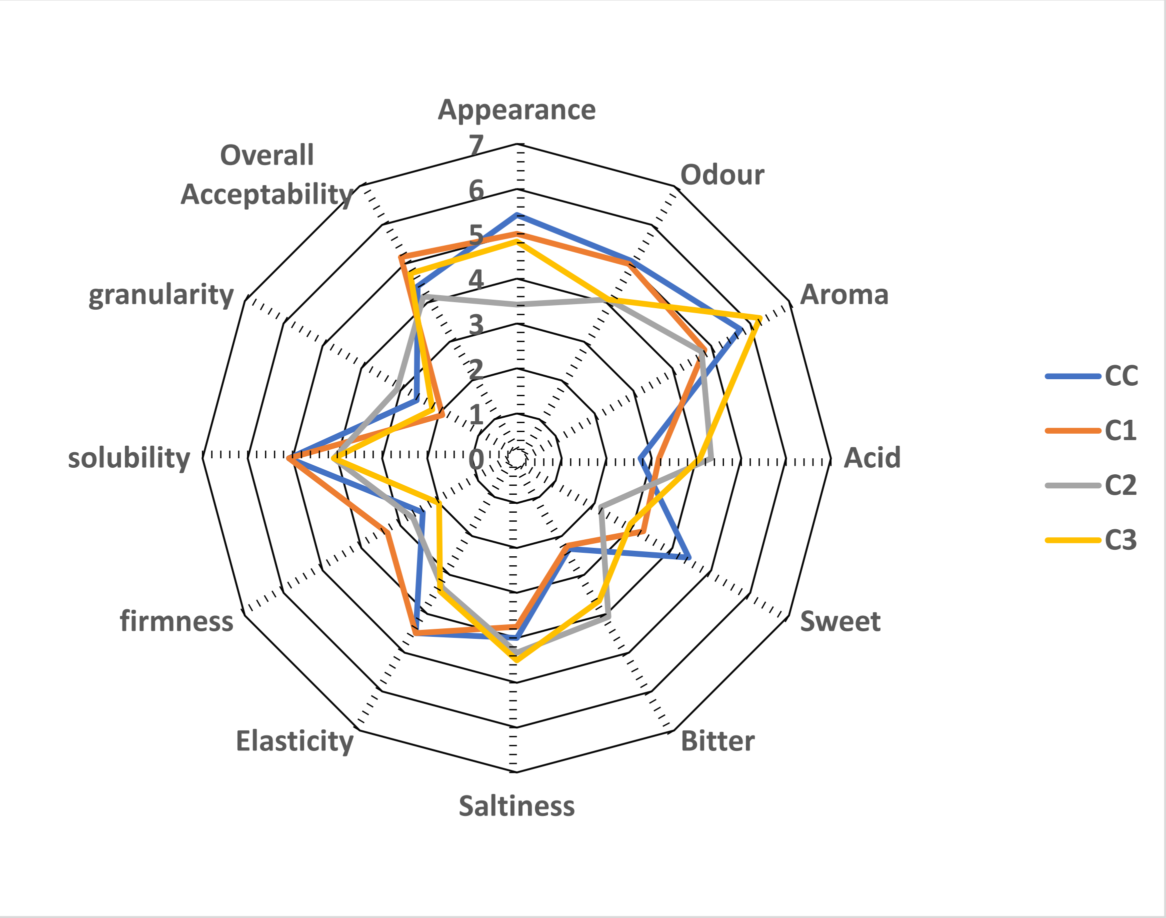

Figure S2. Spider plot for sensory scores obtained for each attribute in control (CC) and probiotic cheeses (C1, C2, C3) at 60 days of ripening.

Figure S3. Values of total saturated fatty acids (SFA) (**A**), monounsaturated fatty acids (MUFA) (**B**), and polyunsaturated fatty acids (PUFA) (**C**), expressed as % of total fatty acids, measured in the control (CC) and probiotic cheeses (C1, C2, C3) at different ripening times by HPLC-ELSD; (n = 6).

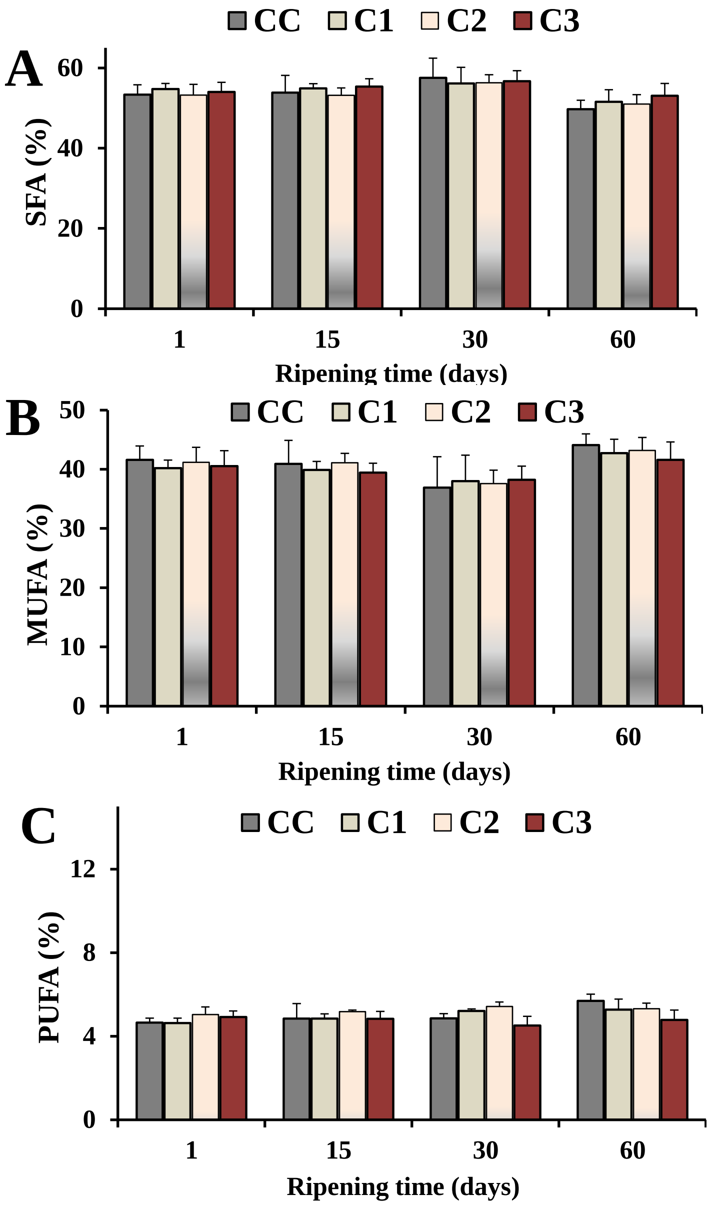

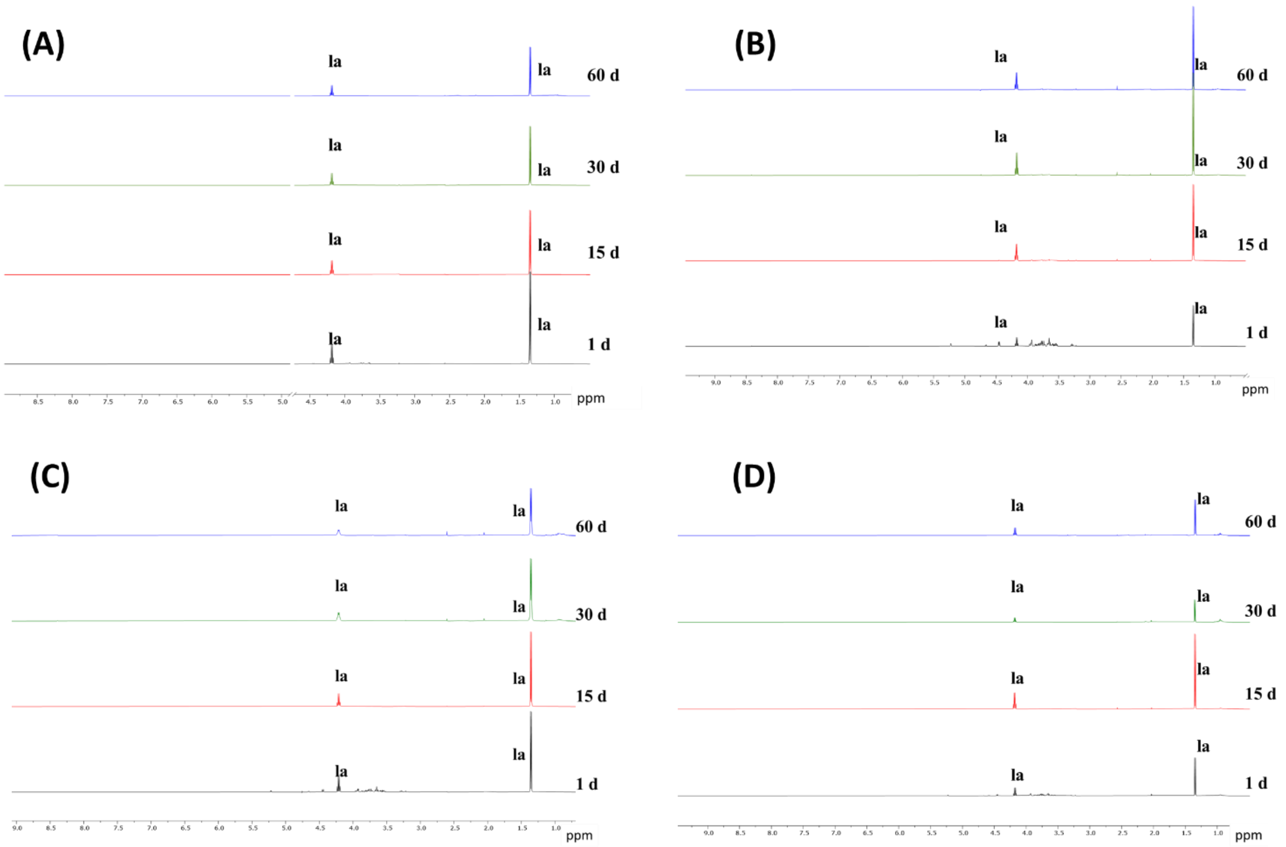

Figure S4. Representative 500 MHz ^{1}H NMR spectra of the aqueous extracts of (A) CC, (B) C1, (C), C2 and (D) C3 Caciotta at different ripening days. Abbreviation: la, lactic acid.

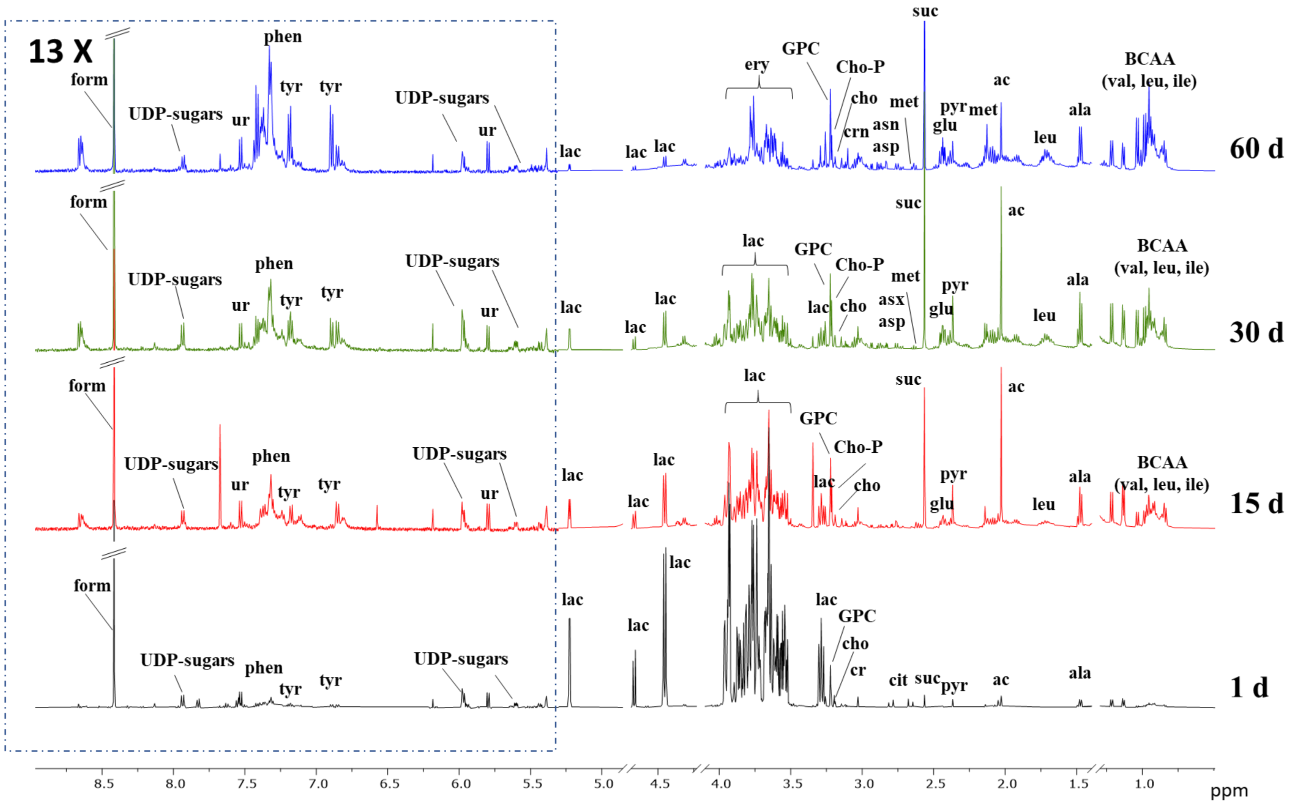

Figure S5. Representative 500 MHz ^{1}H NMR spectra of the aqueous extracts of C1 Caciotta at different ripening days. The peaks of lactic acid have been removed to facilitate the vertical scale increase. Abbreviations: ac, acetic acid; ala, alanine; asp, aspartic acid; asn, asparagine; BCAA, branched-chain amino acids; cit, citric acid; cho, choline; Cho-P, phosphocholine; cr, creatine; crn, creatinine; ery, erythritol; form, formic acid; glu, glutamate; GPC, glycero-phosphocholine; ile; isoleucine; lac, lactose; leu, leucine; met, methionine; phen, phenylalanine; pyr, pyruvic acid; suc, succinic acid; tyr, tyrosine; UDP-sugars, uridine diphosphate-related sugars, ur, uracile; val, valine.

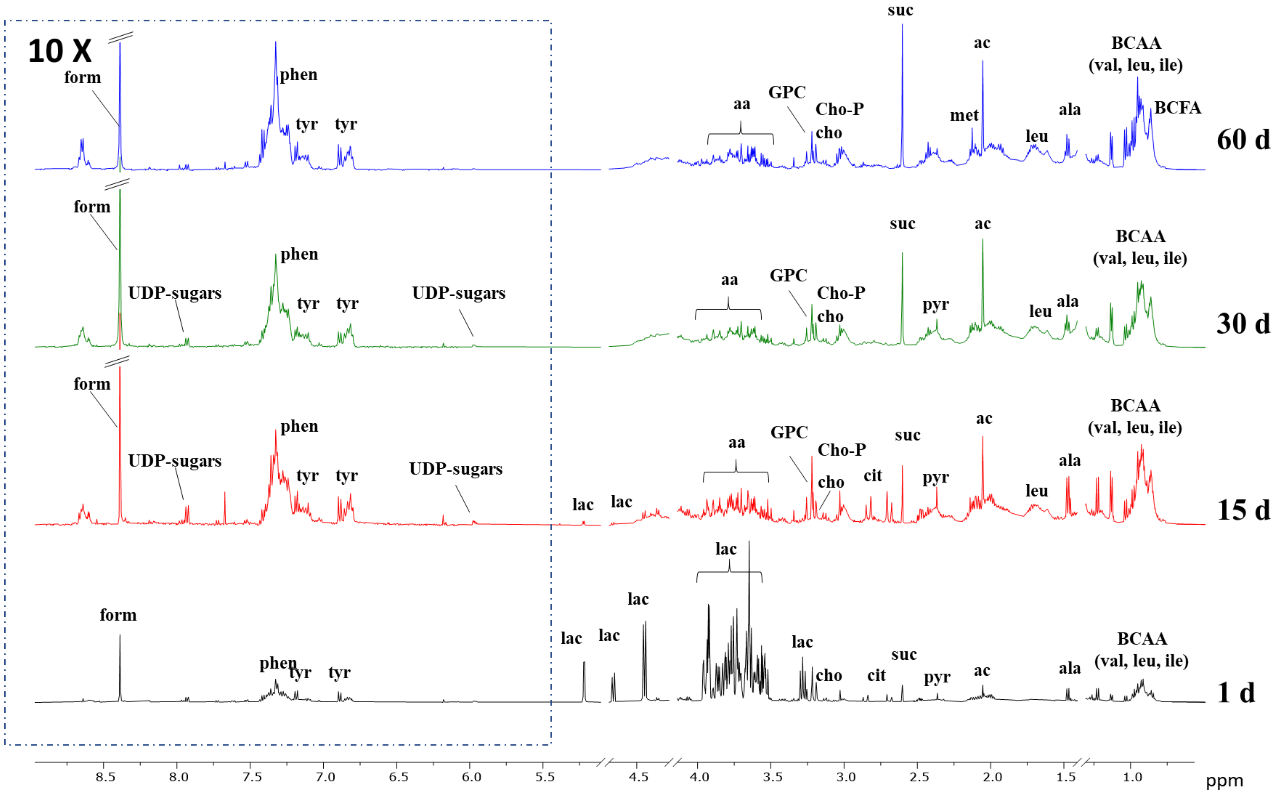

Figure S6. Representative 500 MHz ^{1}H NMR spectra of the aqueous extracts of C2 Caciotta at different ripening days. The peaks of lactic acid have been removed to facilitate the vertical scale increase. Abbreviations: aa, amino acids; ac, acetic acid; ala, alanine; BCAA, branched-chain amino acids; BCFA, branched-chain fatty acids; cho, choline; Cho-P, phosphocholine; cit, citric acid; form, formic acid; GPC, glycero-phosphocholine; ile, isoleucine; lac, lactose; leu, leucine; met, methionine; phen, phenylalanine; suc, succinic acid; tyr, tyrosine; UDP-sugars, uridine diphosphate-related sugars; val, valine.

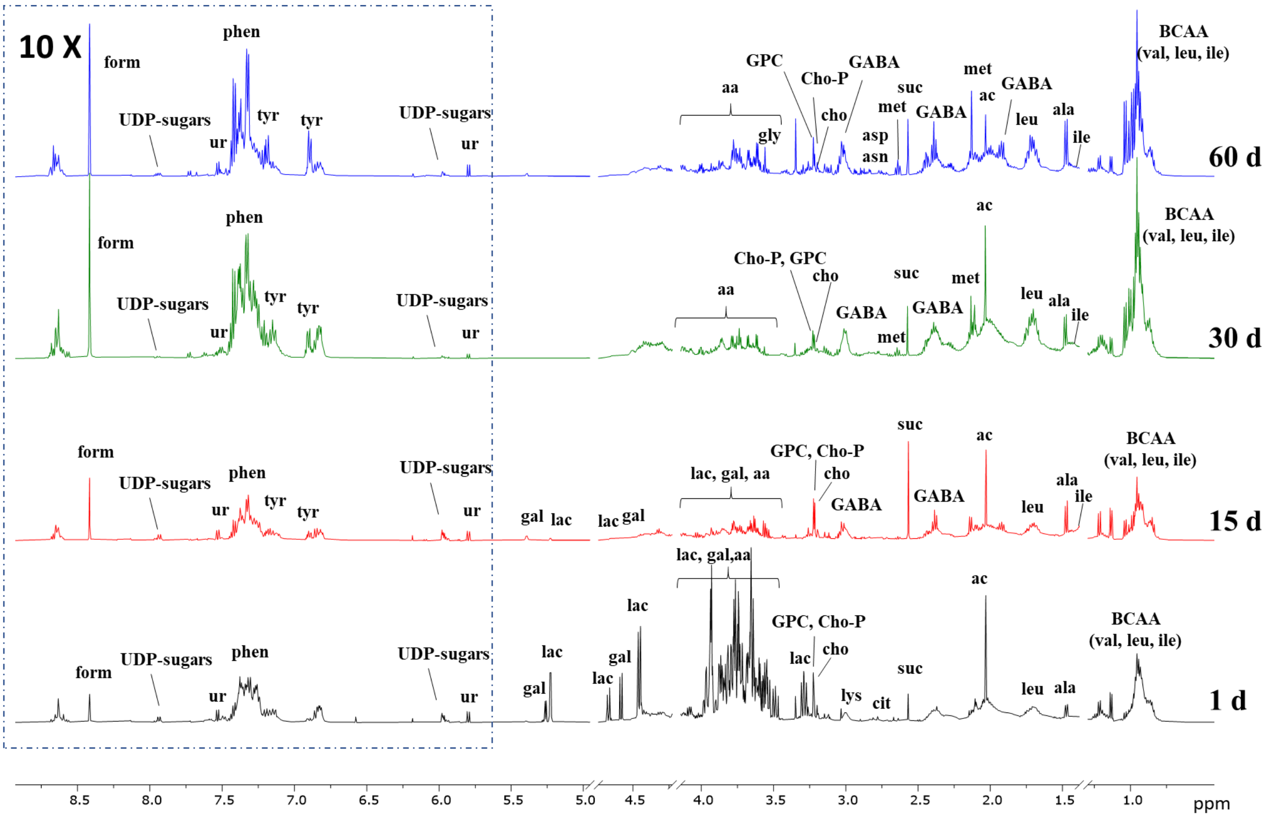

Figure S7. Representative 500 MHz ^{1}H NMR spectra of the aqueous extracts of C3 Caciotta at different ripening days. The peaks of lactic acid have been removed to facilitate the vertical scale increase. Abbreviations: aa, amino acids; ac, acetic acid; ala, alanine; asp, aspartic acid; asn, asparagine; BCAA, branched-chain amino acids; cho, choline; Cho-P, phosphocholine; cit, citric acid; form, formic acid; GABA, γ-aminobutyric acid; GPC, glycero-phosphocholine; ile, isoleucine; lac, lactose; leu, leucine; met, methionine; phen, phenylalanine; suc, succinic acid; tyr, tyrosine; UDP-sugars, uridine diphosphate-related sugars; ur, uracile; val, valine.

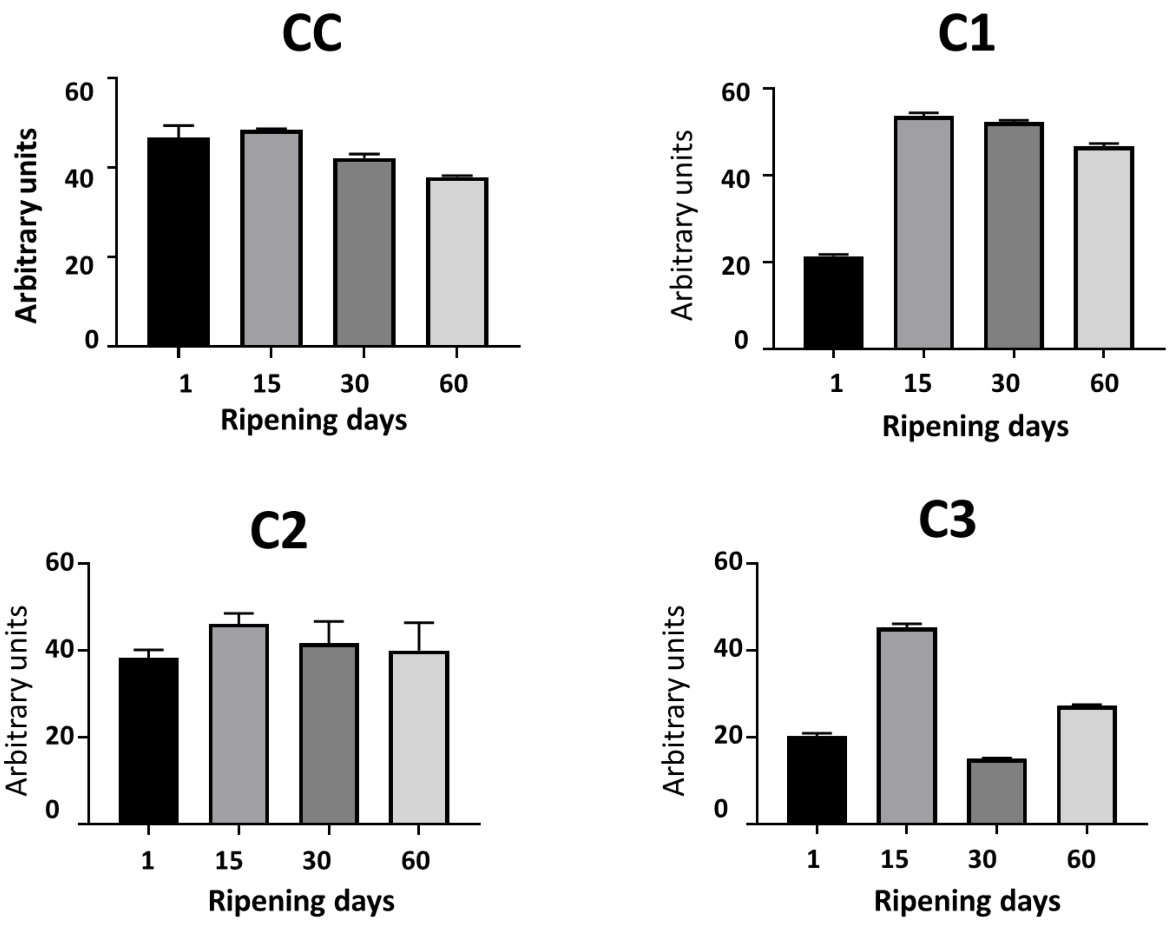

Figure S8. Average levels of lactic acid in the aqueous extract of Caciotta expressed in arbitrary units and calculated by NMR signal integration.

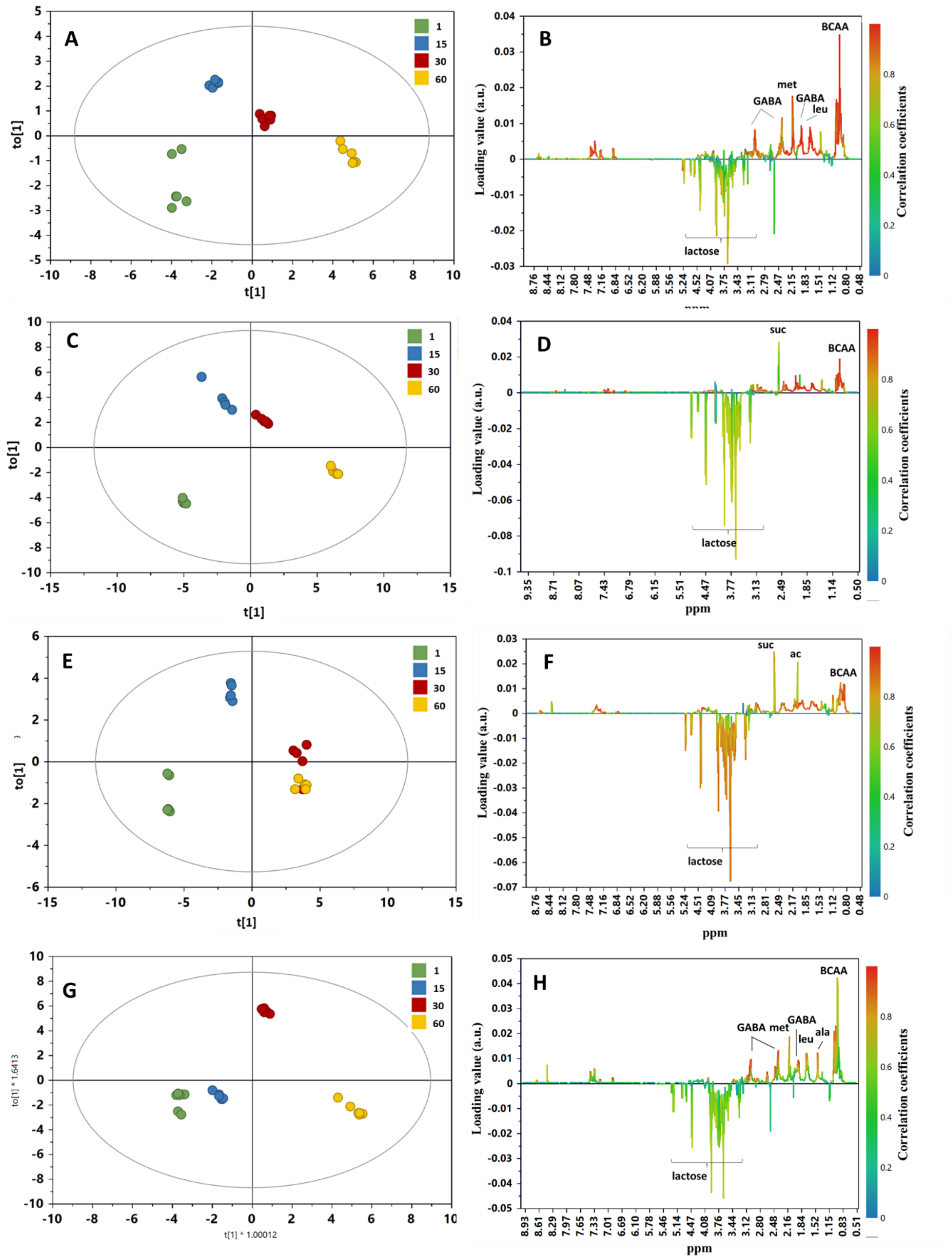

Figure S9. OPLS score (left) and S-line correlation coefficient plots (right) for four types of Caciotta cheese: A-B) CC; C-D) C1; E-F) C2; G-H) C3. Color coded metabolites that significantly correlate with cheese aging (cut-off values: $p(\text{cov}) \geq |0.05|$ and $p(\text{corr}) \geq |0.5|$) are annotated on the S-line plot. Keys: ac, acetic acid; BCAA, branched chain amino acids; GABA, γ-aminobutyric acid; leu, leucine; met, methionine; suc, succinic acid.

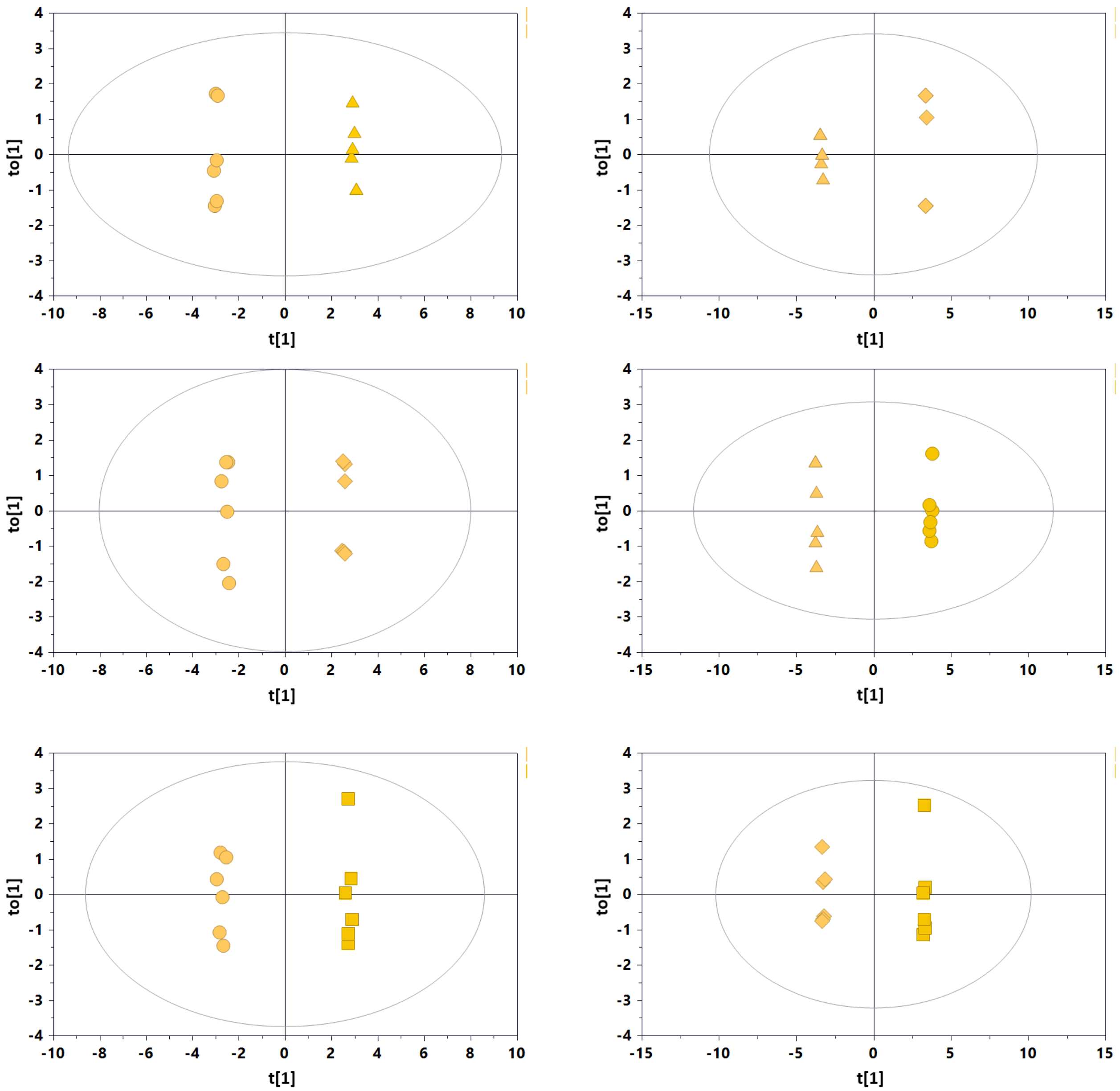

Figure S10. OPLS-DA score plots derived from the pair-wise comparisons between the ^{1}H NMR spectra of the aqueous extract of Caciotta at 60 days of ripening: CC (●), C1 (▲), C2 (♦), C3 (■). For all models $Q^2Y > 0.95$ and CV-ANOVA p-value < 0.0001.
